# Late Cretaceous Paleoceanographic Evolution and the Onset of Cooling in the Santonian at Southern High Latitudes (IODP Site U1513, SE Indian Ocean)

**DOI:** 10.1029/2021PA004353

**Published:** 2022-01-20

**Authors:** Maria Rose Petrizzo, Kenneth G. MacLeod, David K. Watkins, Erik Wolfgring, Brian T. Huber

**Affiliations:** ^1^ Department of Earth Sciences "Ardito Desio" University of Milan Milan Italy; ^2^ Department of Geological Sciences University of Missouri‐Columbia Columbia MO USA; ^3^ Department of Earth and Atmospheric Sciences University of Nebraska Lincoln NE USA; ^4^ Department of Geology University of Vienna Vienna Austria; ^5^ National Museum of Natural History Smithsonian Institution Washington DC USA

**Keywords:** planktonic foraminifera, benthic foraminifera and calcareous nannofossils, oxygen and carbon stable isotopes, paleotemperature, paleoecology and paleoceanography, Santonian cooling, Late Cretaceous

## Abstract

The latest Cenomanian to Santonian sedimentary record recovered at IODP Expedition 369 Site U1513 in the Mentelle Basin (SE Indian Ocean, paleolatitude 60°S at 85 Ma) is studied to interpret the paleoceanographic evolution in the Southern Hemisphere. The planktonic foraminiferal assemblage changes, the depth ecology preferences of different species, and the surface and seafloor temperature inferred from the stable isotopic values measured on foraminiferal tests provide meaningful information to the understanding of the Late Cretaceous climate. The hothouse climate during the Turonian‐Santonian, characterized by weak latitudinal temperature gradients and high atmospheric CO_2_ concentrations, is followed by a progressive cooling during the Campanian. At Site U1513 the beginning of this climatic transition is nicely recorded within the Santonian, as indicated by an ∼1‰ increase in δ^18^O values of planktonic foraminifera suggesting a decline in surface water paleotemperatures of 4°C. The onset of cooling is mirrored by changes in the planktonic foraminiferal assemblages including extinctions among surface and deep dwellers, appearances and diversification of newly evolving taxa, and changes from predominantly epifaunal oxic to infaunal dysoxic/suboxic taxa among co‐occurring benthic foraminifera. Overall, the data presented here document an interval in the Santonian during which the rate of southern high latitude cooling increased. Both surface and bottom waters were affected, although the cooling signal is more evident in the data for surface waters. This pattern of cooling ascribes the deterioration of the Late Cretaceous climate to decreased CO_2_ in the atmosphere and changes in the oceanic circulation correlated with enhanced meridional circulation.

## Key Points

- Latest Cenomanian‐Santonian integrated planktonic foraminifera and calcareous nannofossils biostratigraphy at southern high latitudes (62°S)
- Onset in the Santonian of the Late Cretaceous cooling revealed by foraminifera changes and stable isotopes in the Southern Hemisphere
- The Santonian cooling event affected surface and bottom waters and was more dramatic in the surface waters with paleotemperatures of 14°C

## 1 Introduction

The evolution of Cretaceous climate has been extensively studied through paleontological records, proxy‐based reconstructions, and model simulations of sea‐surface temperatures (e.g., Barron et al., 1995; Craggs et al., 2012; Donnadieu et al., 2006; Hallam, 1985; Hay, 2008; Huber et al., 1995; Otto‐Bliesner et al., 2002; Poulsen et al., 2001; Pucéat et al., 2003). The Cretaceous Period was characterized by a climate with much warmer tropical and polar temperatures than today and weak latitudinal temperature gradients (e.g., Barron, 1983; Huber et al., 1995; Littler et al., 2011; O’Brien et al., 2017) as well as high atmospheric CO_2_ concentrations (e.g., Barclay et al., 2010; Bice et al., 2006; Bice & Norris, 2002; Sinninghe Damsté et al., 2008; Wang et al., 2014). The latter is considered responsible for triggering and maintaining the globally warm climate (e.g., Barron et al., 1995; Crowley & Berner, 2001; Fletcher et al., 2008; Foster et al., 2017; Hay & Floegel, 2012; Royer et al., 2007; Wang et al., 2014). Eustatic sea level during the Cretaceous was higher than present‐day (Haq, 2014; Miller et al., 2005) and permanent ice sheets were absent (e.g., Barron, 1983; Huber et al., 2002; Jenkyns et al., 2004; MacLeod et al., 2013), although small‐scale glaciation events have been proposed (e.g., Bornemann et al., 2008; Ladant & Donnadieu, 2016; Price, 1999; Price & Nunn, 2010). The Cretaceous was also marked by oceanographic changes, such as the opening of the Equatorial Atlantic Gateway (Friedrich & Erbacher, 2006; Wagner & Pletsch, 1999), the enlargement and deepening of the North and South Atlantic basins (Frank & Arthur, 1999), and progressive narrowing of the Tethyan Ocean (Cramer et al., 2009; Friedrich et al., 2012; Jones et al., 1995; Sewall et al., 2007) and Caribbean Gateway (Ladant et al., 2020; MacLeod et al., 2011).

Low to high latitude paleotemperature estimates for the Late Cretaceous, mainly based on global stable oxygen isotopes (δ^18^O) and TEX_86_ compilations, reveal a similar trend that highlights extreme warmth during the Cenomanian‐Turonian, some amelioration but continued warmth through the Coniacian‐Santonian, and progressive cooling extending through the Campanian and into the Maastrichtian (e.g., Ando et al., 2013; Bornemann et al., 2008; Clarke & Jenkyns, 1999; Cramer et al., 2011, 2009; Douglas & Savin, 1975; Friedrich et al., 2012; Huber et al., 2002, 1995, 2018; Jarvis et al., 2011; Jenkyns et al., 1994; Linnert et al., 2018, 2014; MacLeod et al., 2013; Moriya, 2011; Norris & Wilson, 1998; O’Brien et al., 2017; Pucéat et al., 2005; Savin, 1977; Schouten et al., 2003; Scotese et al., 2021; Steuber et al., 2005; Takashima et al., 2006; van Helmond et al., 2014; Voigt et al., 2004). Specifically, the transition from the hothouse of the latest Cenomanian and Turonian into the coolhouse of the late Campanian and Maastrichtian is reported to occur in concert with a number of global changes that affected the ocean‐climate system and Earth’s biota. These include: (a) large‐scale tectonic changes including the expansion of the Atlantic and Southern Ocean (e.g., Sewall et al., 2007); (b) decreasing atmospheric CO_2_ levels (e.g., Barron et al., 1995; Fletcher et al., 2008; Tabor et al., 2016; Wang et al., 2014) probably associated with continental volcanism and/or with reduced CO_2_ flux from mid‐ocean ridge volcanism and arc magmatism (e.g., Berner, 2004; Berner et al., 1983; Cogné & Humler, 2006; Kent & Muttoni, 2013; McKenzie et al., 2016; van der Meer et al., 2014); (c) radiation of angiosperms (e.g., Boyce et al., 2009); (d) opening of the Equatorial Atlantic Gateway and northward intrusion of cooler Atlantic water masses from higher southern latitudes (Forster et al., 2007; Frank & Arthur, 1999; Friedrich & Erbacher, 2006; Friedrich et al., 2012; MacLeod & Huber, 1996); (e) formation of deep‐ and/or intermediate‐water at high latitudes and reduction of warm and saline water masses in the subtropics (Barrera et al., 1997; Barrera & Savin, 1999; Friedrich et al., 2009, 2012; MacLeod, 1994; MacLeod et al., 2005, 2011; MacLeod & Huber, 2001) which coincide with a major reorganization of oceanic circulation during the Santonian‐Campanian evidenced by neodymium isotopic data (Haynes et al., 2020; MacLeod et al., 2011; Murphy & Thomas, 2012; Robinson et al., 2010; Robinson & Vance, 2012); and (f) increasing continental weathering and erosion resulting from enhanced CO_2_ consumption by silicate weathering (Chenot et al., 2018). The occurrence of ephemeral ice sheets (Stoll & Shrang, 2000) and glacio‐eustasy (Miller et al., 2005) have been invoked as possible mechanisms responsible for sea level changes despite evidence for global warmth. The complex interaction of the mechanisms listed above make determining the ultimate cause of the long‐term Late Cretaceous cooling uncertain.

Paleogeographic changes have been invoked as having a significant influence on global temperature evolution across the Late Cretaceous (Donnadieu et al., 2006; Fluteau et al., 2007; Frank & Arthur, 1999; Haynes et al., 2020; Ladant & Donnadieu, 2016; Poulsen et al., 2003). However, there is a lack of consensus among climate model results. Some model simulations indicate a minor impact of paleogeographic changes on the Late Cretaceous cooling suggesting that a decrease in CO_2_ concentration was the main driver (Tabor et al., 2016) in agreement with paleotemperatures proxies (Haynes et al., 2020; Linnert et al., 2014; Pucéat et al., 2007) and CO_2_ reconstructions (Fletcher et al., 2008; Wang et al., 2014). Some other models (Donnadieu et al., 2016; Ladant et al., 2020; Lunt et al., 2016) and proxy‐based evidence (e.g., Friedrich et al., 2012; Huber et al., 2018; MacLeod et al., 2011; Martin et al., 2012; Murphy & Thomas, 2013; Robinson et al., 2010; Robinson & Vance, 2012) suggest an important role of paleogeographic reorganization. In these studies geometry and depth of oceanic basins and gateways coupled with decreased CO_2_ are considered to be responsible for changes in oceanic circulation, particularly increasing importance of high latitude sources of intermediate and deep waters, that paced the long‐term cooling in the Late Cretaceous.

In terms of planktonic foraminiferal evolution, the climatic transition from the hot to cool climate in the Santonian‐early Campanian coincides with a remarkable compositional change characterized by taxonomic diversification followed by extinctions. A 3 My‐long major foraminiferal turnover is documented in the Coniacian‐Santonian interval during which pre‐Campanian keeled taxa became extinct and were replaced by newly evolved late Santonian‐early Campanian taxa. The assemblage changes (also known as the Santonian turnover: Petrizzo et al., 2017) is registered worldwide (e.g., Caron & Homewood, 1983; Hart, 1999; Hart & Bailey, 1979; Petrizzo, 2002; Premoli Silva & Sliter, 1999; Wonders, 1980), although it is better documented and resolved at low and mid‐latitudes than at high latitudes at least in part because the low to mid‐latitude assemblages have a higher diversity and more species changes compared to the record at high latitudes (Petrizzo et al., 2020). Thus, the potential signal is higher.

Hypotheses proposed to explain the Santonian foraminiferal turnover include: (a) tectonically forced changes in ocean circulation (Ando et al., 2013; Premoli Silva & Sliter, 1999); (b) the onset of the Late Cretaceous cooling trend during the late Santonian (Petrizzo, 2002) combined with species competition within particular depth habitats (Falzoni et al., 2016); and (c) the development of regional anoxic events (i.e., Oceanic Anoxic Event 3; e.g., Arthur & Schlanger, 1979; Jenkyns, 1980; Ryan & Cita, 1977; Schlanger & Jenkyns, 1976; Wagreich, 2012) in the Atlantic and adjacent epicontinental sea resulting in the enlargement of ecological niches in the more oxygenated Tethys, Pacific and Indian oceans (Wagreich, 2009).

**Figure 1:**
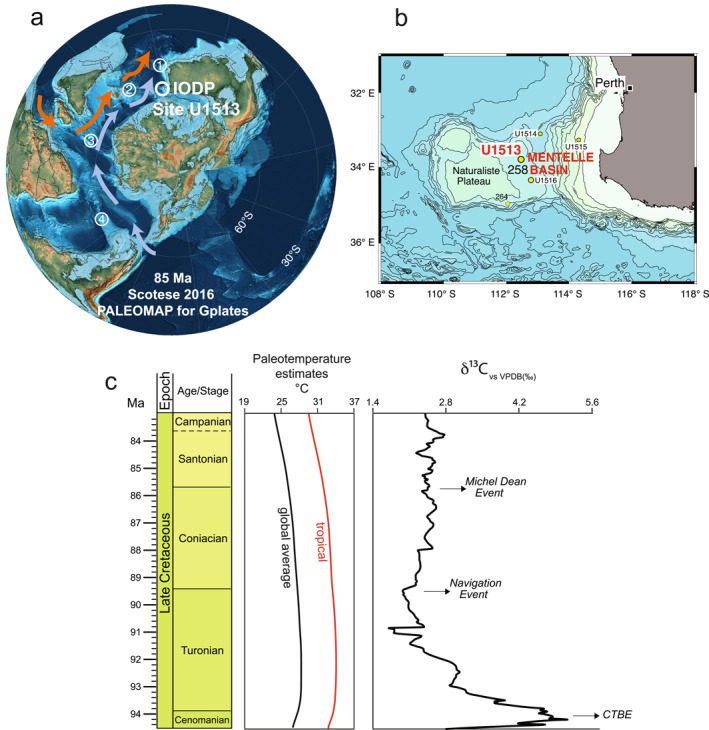
(a) Paleogeographic reconstruction (Scotese, 2016) for the Santonian (85 Ma), with location of International Ocean Discovery Program (IODP) Expedition 369 Site U1513 and of the other localities mentioned in the text: 1—Exmouth Plateau, 2—Kerguelen Plateau, 3—Northeast Georgia Rise, 4—Falkland Plateau. The paleolatitude of Site U1513 is calculated as 59.7°S at 85 Ma using paleolatitude.org (van Hinsbergen et al., 2015). The inferred subtropical gyre (orange arrows) and the subantarctic gyre (light blue arrows) are shown, see explanation in the text. (b) Location of the sites drilled during IODP Expedition 369 (U1513, U1514, U1515, and U1516) and nearby Deep Sea Drilling Project (DSDP) Sites 258 and 264 (modified after Huber et al., 2019a). (c) Paleotemperature estimates (Scotese et al., 2021) and carbon isotope references curve (Cramer & Jarvis, 2020) for the Turonian‐Santonian interval; chronostratigraphy according to GTS 2020 (Gradstein et al., 2020). *Abbreviations*: CTBE, Cenomanian/Turonian Boundary Event.

This study is focused on the latest Cenomanian to Santonian sedimentary sequence recovered at Site U1513 during International Ocean Discovery Program (IODP) Expedition 369. The site is located in the Mentelle Basin (eastern flank of the Naturaliste Plateau, SE Indian Ocean, SW Australia) and was at a paleolatitude calculated as ranging from 57°S to 62°S during the Late Cretaceous (Hay et al., 1999; Müller et al., 2016; Scotese, 2016; van Hinsbergen et al., 2015; Figure 1a). We investigate the foraminiferal record to interpret the paleoceanographic changes and the paleotemperature evolution from the thermal maximum across the Cenomanian‐Turonian interval followed by progressive cooling through the Santonian. In the present study, quantitative foraminiferal assemblage data and depth habitat ecologies based on species‐specific stable isotope analyses are used to interpret the paleoceanographic changes and the history of surface water circulation in the southern high latitudes. Paleotemperature estimates derived from foraminiferal and bulk carbonate stable isotopic measurements provide information on the pattern of the climatic transition from the hothouse to the coolhouse which are crucial to an accurate understanding of the evolution of the circum‐Antarctic climate.

## 2 Materials and Methods

**Figure 2:**
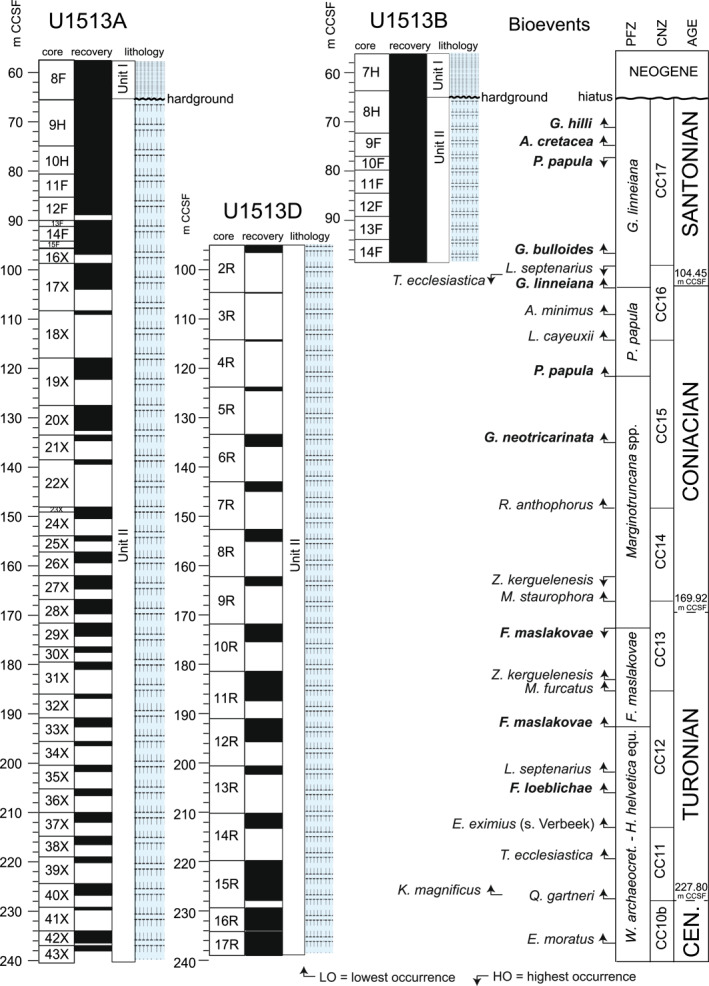
Holes U1513A, U1513B, and U1513D: planktonic foraminifera and calcareous nannofossil bioevents. Core recovery and lithologic units according to Huber et al. (2019a). Age: the Cenomanian/Turonian boundary is approximated at the lowest occurrence of *Quadrum gartneri* (dashed line); the Turonian/Coniacian boundary is tentatively placed at the midpoint between the highest occurrence of *Falsotruncana maslakovae* and the lowest occurrence of *Micula staurophora* (dashed line); and the Coniacian/Santonian boundary is identified at the lowest occurrence of *Globotruncana linneiana,* secondary criterion for the identification of the Santonian GSSP (solid line). Planktonic foraminiferal biozonation follows Robaszynski & Caron (1995) and Petrizzo et al. (2020). Calcareous nannofossil biozonation follows Perch‐Nielsen (1985). Planktonic foraminiferal bioevents are in bold. *Abbreviations:* m CCSF, core composite depth below sea floor in meters; CNZ, calcareous nannofossils zones; PFZ, planktonic foraminifera zones. See text for further explanation.

IODP Site U1513 (33°47.6084S, 112°29.1338E) lies at 2,800 m water depth on the western margin of the Mentelle Basin located on the eastern flank of the Naturaliste Plateau and off the south western margin of Australia (SE Indian Ocean; Figure 1a). This study focuses on the latest Cenomanian to Santonian sedimentary record recovered from Holes U1513A, U1513B, and U1513D whose overlapping portions together provide relatively continuous recovery of this interval (Figure 2). Site U1513 is located 1.1  km east‐northeast of Deep Sea Drilling Project (DSDP) Leg 26 Site 258 (Figure 1b), which was spot‐cored (20% recovery) in the Late Cretaceous interval (Luyendyk & Davies, 1974). The objectives at Site U1513 were to improve recovery within gaps of the record at Site 258, to recover a complete record of Oceanic Anoxic Event 2 (OAE 2), and to sample unaltered basalts from the basement of the Naturaliste Plateau.

The Late Cretaceous sedimentary record is separated from the overlying Miocene sediments by a 40 cm‐thick complex of hardgrounds with at least two distinct mineralized surfaces that were recovered in the intervals U1513A‐9H‐1, 0–10  cm and U1513B‐8H‐2, 7–47  cm, although the features are best seen in the latter. A 182.93 m‐thick Upper Cretaceous sedimentary record was recovered beneath the hardground complex (Figure 2). The stratigraphic succession is assigned to lithostratigraphic Unit II which is composed of white to greenish gray calcareous and nannofossil ooze and chalk, clayey nannofossil chalk, and silicified limestone that becomes generally more clay rich down core. The bottom of the studied stratigraphic succession (U1513A‐43X and U1513D‐17R) is about 3 m above a 2.3 m‐thick bed of mottled grayish green claystone that has a sharp basal contact with a bed of black claystone assigned to lithostratigraphic Unit III. Investigating these grayish and black lithologies characterized by low carbonate content (CaCO_3_ <0.5  wt%), that were interpreted as the sedimentary expression of Oceanic Anoxic Event 2 (OAE 2) by Huber et al. (2019a), is beyond the scope of this study and will be the subject of future publications.

Data from Holes U1513A, U1513B, and U1513D are plotted on the CCSF depth scale (Core Composite Depth below Sea Floor, equivalent to mcd, meters composite depth; Huber et al., 2019b; LIMS online report portal at http://web.iodp.tamu.edu/LORE/). The chronostratigraphy is according to Gradstein et al. (2020).

For micropaleontological analysis, 145 rock samples were dried, weighed, soaked in a solution of water and Hydrogen Peroxide (H_2_O_2_), washed over 250, 125, and 38  μm sieves, and dried. Radiolaria and foraminifera genera and species were counted in splits for each size fraction of these washed residues. The number of specimens for each category were calculated from the number of specimens counted and the fraction of the total counted split represented; the total number of specimens were obtained for each sample by adding the values of specimens/category for all the size fractions examined. Absolute abundances of microfossils were calculated as the number of specimens per gram of dry sediment. Planktonic foraminiferal taxonomy (Supporting  Information S1) follows the pforams@mikrotax database at http://www.mikrotax.org/pforams (Huber et al., 2016). The planispiral species traditionally assigned to the genus *Globigerinelloides* are grouped together (=planispiral taxa) because this group is currently under taxonomic revision as the genus has been shown to be polyphyletic (see discussion in Petrizzo et al., 2017). The planktonic foraminiferal biozonation follows Robaszynski & Caron (1995), Premoli Silva & Sliter (1995), Petrizzo (2003), and Petrizzo et al. (2020).

Benthic foraminifera counts were made using the >125  μm size fraction; specimens present in size fractions between 125 and 38  μm were excluded because of the difficulty in confidently identifying juvenile individuals in these small size fractions. Benthic foraminiferal taxonomy follows the references listed in Supporting  Information S1. Benthic foraminiferal taxa were tallied as either infaunal and epifaunal taxa, and also as oxic, dysoxic, or suboxic/anoxic taxa according to their environmental preferences following the classifications by Alegret et al. (2003), Alegret & Thomas (2007), Murray (2006), Jorissen et al. (2007), and references therein. The changes in benthic foraminiferal assemblages for oxic, dysoxic, and suboxic groups of taxa were analyzed using the statistics package of R (R Core Team, 2021) to calculate a smoother LOWESS curve (span  =  0.2) through the data.

Epifaunal benthic foraminifera are similar to recent analogs and mostly characterized by taxa having planispiral or trochospiral chamber arrangements such as *Notoplanulina, Nuttallinella, Cibicides, Gavelinella, Eponides*, and *Gyroidinoides*. The latter genus is also known as an opportunistic taxon with unclear habitat preference being either considered an infaunal or epifaunal taxon and even tolerant to anoxic environments (Alegret et al., 2003; Friedrich et al., 2006; Jorissen et al., 2007). Agglutinated benthic foraminifera (*Dorothia, Gaudryina*), calcareous elongated uniserial and flattened benthic foraminifera (*Dentalina, Nodosaria*, *Planularia*, *Pleurostomella*), lenticulinid forms (*Lenticulina*, *Astacolus*), and polymorphinid foraminifera (*Ellipsoglandulina, Ellipsoidella*) are inferred to prefer an infaunal habitat. Most epifaunal benthic foraminiferal taxa recorded at Site U1513 share a preference for well oxygenated habitats. Taxa inferred as tolerant to anoxic conditions include agglutinated taxa (*Dorothia, Ammodiscus*) as well as polymorphinid foraminifera and pleurostomellids (e.g., *Ellipsoglandulina, Ellipsoidella, Glandulina*). Taxa considered tolerant of suboxic habitats include lagenids, vaginulinids, *Dentalina*, and *Nodosaria*.

Biostratigraphic analysis of calcareous nannofossils was performed on 123 samples from Holes U1513A, U1513B, and U1513D using smear slides prepared following Watkins & Bergen (2003). Presence/absence data for taxa encountered for each sample is tabulated in data table at https://doi.pangaea.de/10.1594/PANGAEA.939392. Calcareous nannofossil biostratigraphy follows Perch‐Nielsen (1985).

Foraminiferal isotope data (δ^13^C and δ^18^O) were obtained from single species separates of well‐preserved planktonic and benthic foraminifera picked from samples where sufficient number of such individuals were present. Species were selected according to their abundance and degree of preservation, and only specimens showing no visible diagenetic calcite and infilled sparry calcite in the chambers or minor test wall recrystallization were measured. Material for bulk analyses were milled from cleaned surfaces of sediment samples. All analyses were made at the University of Missouri using a Kiel III carbonate device and Delta Plus IRMS. External precision calculated as 1 standard deviation of the uncorrected repeated analyses of the standard NBS‐19 run every seventh or eighth sample on average throughout the course of the study, and that value was ±0.03‰ and ±0.08‰, for δ^13^C and δ^18^O, respectively. Internal standards (fine fraction separates of Cretaceous chalk) are used to monitor machine performance before each run is started and at the start of each set of 46 analyses. To reduce effects of day‐to‐day variation, analyses within each run were corrected by the difference between the average values measured for the NBS‐19 during the run (typically 6) and the nominal values of 1.95‰ (δ^13^C) and −2.20‰ (δ^18^O) for the standard. Results are reported on the Vienna‐PDB scale. Paleotemperature estimates at Site U1513 are calculated from the foraminiferal δ^18^O values, assume seawater δ^18^O of −1‰_SMOW_ and use the paleotemperature equation of Kim & O’Neil (1997) reformulated by Bemis et al. (1998) following the assumptions detailed in Huber et al. (2018).

## 3 Calcareous Plankton Bioevents and Integrated Biostratigraphy

The studied stratigraphic interval at Site U1513 spans from the uppermost Cenomanian to the upper Santonian according to planktonic foraminiferal and calcareous nannofossil biostratigraphy. The planktonic foraminiferal biozonation for southern mid‐ to high latitudes by Petrizzo et al. (2020) is applied and correlated with calcareous nannofossil CC zones of Perch‐Nielsen (1985) to provide an integrated calcareous plankton biozonation scheme at about 60°S paleolatitude. Lowest and highest occurrences of calcareous plankton events, including those that mark zonal boundaries, are shown in Table 1 and Figure 2.

**Table 1:** Planktonic Foraminifera and Calcareous Nannofossil Bioevents at Site U1513

Sample	Depth m CCSF	Bioevents
U1513B‐8H‐2, 7–47 cm	64.98	hardground
U1513B‐8H‐5, 98–100 cm	70.43	**LO *Globotruncana hilli***
U1513B‐9F‐3, 30–32 cm	75.29	**LO *Archaeoglobigerina cretacea***
U1513B‐10F‐1, 50–52 cm	77.60	**HO *Planoheterohelix papula***
U1513A‐15F‐CC, 0–10 cm	95.60	LO *Calculites obscurus*
U1513A‐16X‐1, 30–33 cm	97.26	**LO *Globotruncana bulloides***
U1513A‐17X‐1, 50–53 cm	99.20	HO *Lithastrinus septenarius*
U1513A‐17X‐2, 88–90 cm	101.08	HO *Thiersteinia ecclesiastica*
U1513A‐17X‐3, 80–83 cm	104.45	**LO *Globotruncana linneiana***
U1513A‐18X‐CC, 0–5 cm	108.64	LO *Amphizygus minimus*
U1513A‐18X‐CC, 0–5 cm	108.64	LO *Lithastrinus grillii*
U1513D‐4R‐CC, 0–1 cm	114.30	LO *Lucianorhabdus cayeuxii*
U1513D‐4R‐CC, 0–1 cm	114.30	LO *Prediscosphaera desiderograndis*
U1513A‐19X‐3, 75–78 cm	121.66	**LO *Planoheterohelix papula***
U1513D‐6R‐2, 60–63 cm	135.42	**LO *Globotruncana neotricarinata***
U1513A‐23X‐1, 10–13 cm	148.20	LO *Reinhardtites anthophorus*
U1513A‐27X‐1, 8–11 cm	162.08	HO *Zeugrhabdotus kerguelensis*
U1513A‐28X‐1, 18–21 cm	166.98	LO *Micula staurophora*
U1513A‐29X‐1, 127–130 cm	172.87	**HO *Falsotruncana maslakovae***
U1513D‐11R‐2, 40–43 cm	183.06	LO *Reinhardtites biperforatus*
U1513D‐11R‐2, 40–43 cm	183.06	LO *Zeugrhabdotus kerguelensis*
U1513D‐11R‐4, 45–48 cm	185.61	LO *Marthasterites furcatus*
U1513A‐33X‐1, 10–13 cm	193.62	**LO *Falsotruncana maslakovae***
U1513D‐13R‐CC, 17–18 cm	202.25	LO *Lithastrinus septenarius*
U1513A‐36X‐1, 86–89 cm	206.06	**LO *Falsotruncana loeblichae***
U1513A‐37X‐2, 111–114	211.72	**LO *Marginotruncana pseudolinneiana***
U1513D‐14R‐CC, 13–18 cm	213.22	LO *Eiffellitus eximius* (s. Verbeek)
U1513A‐39X‐1, 40–43 cm	219.40	LO *Thiersteinia ecclesiastica*
U1513A‐40X‐2, 80–83 cm	227.13	LO *Kamptnerius magnificus*
U1513D‐15R‐CC, 7–12 cm	227.80	LO *Quadrum gartneri*
U1513D‐17R‐2, 120–123 cm	236.65	LO *Eprolithus moratus*
U1513A‐42X‐2, 37–40 cm	236.45	**LO *Dicarinella hagni***
U1513A‐43X‐1, 35–38 cm	239.06	**LO *Whiteinella brittonensis***

*Note*. Planktonic foraminiferal bioevents are in bold. m CCSF, core composite depth below sea floor in meters; HO, highest occurrence; LO, lowest occurrence.

The lowest occurrence (LO) of *Quadrum gartneri* at 227.80 m CCSF defines the base of Zone CC11 and is a consistently reliable datum for approximating the base of the Turonian Stage as it occurs slightly above the stage criterion in the Global Stratotype Section and Point (GSSP) type section (Kennedy et al., 2000, 2005) and in many other localities worldwide (e.g., Corbett et al., 2014; Dickson et al., 2017; Gale et al., 2020, 2019; Gradstein et al., 2012; Linnert et al., 2011, 2010; Petrizzo et al., 2021; Tsikos et al., 2004; von Salis, 1998). Nannofossil assemblages in the interval below are assigned to Zone CC10b, yield *Eprolithus moratus* at 236.65 m CCSF and are cosmopolitan in nature with only very rare specimens of species considered to be high latitude taxa. Nannofossil Zone CC11 contains two important biostratigraphic events: the LOs of *Kamptnerius magnificus* at 227.13 m CCSF and *Thiersteinia ecclesiastica* at 219.40 m CCSF. In addition to the austral species *T. ecclesiastica*, the high latitude species *Seribiscutum primitivum* and *Repagulum parvidentatum* become common elements of these assemblages at Site U1513 in this zone. The LO of *Eiffellithus eximius* (sensu Verbeek) at 213.22 m CCSF is used to determine the base of the overlying Zone CC12, following the criteria proposed in Gaer & Watkins (2020). At Site U1513, this zone contains the LO of *Lithastrinus septenarius* at 202.25 m CCSF using the criteria of Corbett & Watkins (2014) to differentiate this taxon from its ancestor *Eprolithus moratus*.

The planktonic foraminifera *Rotalipora cushmani* and *Helvetoglobotruncana helvetica* whose highest occurrence (HO) and LO, respectively, are used to constrain and approximate the position of the Cenomanian/Turonian boundary at low to mid‐latitudes (e.g., Caron et al., 2006; Desmares et al., 2007; Elderbak & Leckie, 2016; Eldrett et al., 2015; Falzoni et al., 2018, 2016; Falzoni & Petrizzo, 2020; Gradstein et al., 2012; Keller et al., 2001; Kennedy et al., 2005, 2000; Paul et al., 1999). These species are absent from the Mentelle Basin sediments both at Site U1513 and nearby Site U1516 (Petrizzo et al., 2021). The planktonic foraminifera assemblages from the base of the studied stratigraphic interval (U1513A‐43X and U1513D‐17R) to the LO of *Falsotruncana maslakovae* at 193.62 m CCSF are characterized by low diversity and are dominated by whiteinellids (*Whiteinella baltica* and *Whiteinella brittonensis*), muricohedbergellids, and a few keeled species of *Marginotruncana* and *Dicarinella*. For these reasons, and because no species with remarkable or short stratigraphic ranges have been found, this stratigraphic interval has been considered equivalent to the combined low latitude *Whiteinella archaeocretacea* and *H. helvetica* Zones (Premoli Silva & Sliter, 1995; Robaszynski & Caron, 1995) based on the composition of the assemblages (and despite the absence of the Tethyan biozonal species). *Falsotruncan*a *loeblichae* is recorded at 206.06 m CCSF preceding the lowest occurrence of *F. maslakovae* in agreement with its stratigraphic distribution at low latitudes (Tunisia: Caron, 1981; Robaszynski et al., 1993) and mid‐to high latitudes (Exmouth Plateau, NW Australia, 47°S paleolatitude: Petrizzo, 2000; Kerguelen Plateau, southern Indian Ocean, 50°S paleolatitude: Petrizzo, 2001).

The Turonian/Coniacian boundary, ratified in May 2021 by the International Union of Geological Sciences (IUGS), is defined by the first appearance of the inoceramid bivalve species *Cremnoceramus deformis erectus* and complemented by the Navigation Carbon Isotope Event (Walaszczyk et al., 2021). In the stratotype section (Salzgitter‐Sadler, Germany) and in the auxiliary sections of the GSSP there are no reliable calcareous nannofossil biohorizons closely associated with the Turonian/Coniacian boundary, although the boundary lies within Zone CC13 (Jarvis et al., 2021; Lees, 2008; Sikora et al., 2004; Voigt et al., 2021; Walaszczyk et al., 2021). Accordingly, at Site U1513 the Turonian/Coniacian boundary is inferred to fall within Zone CC13 between the LO of *Marthasterites furcatus* at 185.61 m CCSF and the LO of *Micula staurophora* at 166.98 m CCSF. The LO of the austral species *Zeugrhabdotus kerguelenesis* coincides with that of *Reinhardtites biperforatus* (=*Zeugrhabdotus biperforatus* of some authors) which has its LO at 183.06 m CCSF. The close stratigraphic association of *M. furcatus*, *Z. kerguelenensis*, and *R. biperforatus* is consistent with the literature reports (e.g., Bergen & Sikora, 1999; Burnett, 1998).

The absence at Site U1513 of the planktonic foraminifera *Dicarinella concavata*, whose LO occurs slightly below the stage criterion in the auxiliary sections of the GSSP (Walaszczyk et al., 2021), prevents the precise identification of the base of the Coniacian Stage. Therefore, in this study the Turonian/Coniacian boundary is tentatively placed at the midpoint (169.92 m CCSF) between the HO of the planktonic foraminifera *F. maslakovae* (172.87 m CCSF) and the LO of the calcareous nannofossil *M. staurophora*, which defines the base of Zone CC14 Zone following correlations with the record from Tanzania and Exmouth Plateau (NW Australia, ODP Hole 762) where the two taxa are recorded in the same stratigraphic order (Huber et al., 2017). According to this correlation the *F. maslakovae* Total Range Zone is entirely comprised within the upper Turonian (Petrizzo et al., 2020).

The Coniacian at Site U1513 appears to be completely represented. Nannofossil Zone CC14 is defined by the LO of *M. staurophora* at 166.98 m CCSF and the LO of *Reinhardtites anthophorus* at 148.20 m CCSF. This zone includes the HO of *Z. kerguelenesis* at 162.08 m CCSF, a placement that agrees precisely with that of Burnett (1998). The appearance of austral taxa, such as *Biscutum notaculum* and *Biscutum dissimilis*, in addition to the consistent common abundance of *R. parvidentatum* and the persistent presence of *S. primitivum*, indicates the clearly austral nature of these assemblages. The overlying Coniacian calcareous nannofossils Zone CC15 extends from the LO of *R. anthophorus* to the LO of *Lucianorhabdus cayeuxi* at 114.30 m CSSF, the latter marking the base of Zone CC16.

The stratigraphic interval from the HO of *F. maslakovae* to the LO of *Planoheterohelix papula* (121.66 m CCSF) is assigned to the *Marginotruncana* spp. Zone (Petrizzo, 2000; Petrizzo et al., 2020). The planktonic foraminifera *Globotruncana neotricarinata*, first recorded at 135.42 m CCSF, is a cosmopolitan species with a diachronous range (Petrizzo et al., 2011) which is confirmed at Site U1513 where it precedes the appearance of *P. papula* whereas it first occurs near the top of the *P. papula* Zone in the Exmouth Plateau record at 47°S paleolatitude (Petrizzo, 2000; Petrizzo et al., 2020).

The Coniacian/Santonian boundary is placed at the LO of *Globotruncana linneiana* (104.45 m CCSF), the secondary criterion for the identification of the Santonian GSSP which falls 10  cm above the first occurrence of the primary marker, the inoceramid *Cladoceramus undulatoplicatus* in the GSSP stratotype section at Olazagutia (Spain; Lamolda et al., 2014). This assignment is confirmed by correlation of the sequence of calcareous nannofossils bioevents documented in the stratotype section of the Coniacian/Santonian boundary where the boundary lies within Zone CC16 and between the LO *L. cayeuxii* and the LO of *G. linneiana*.

The interval from the LO of *G. linneiana* to the top of the Cretaceous sediments is assigned to the *G. linneiana* Zone (Petrizzo et al., 2020), and registers a sequence of appearances (*Globotruncana bulloides* at 97.26 m CCSF, *Archaeoglobigerina cretacea* at 75.29 m CCSF, and *Globotruncana hilli* at 70.43 m CCSF) and the disappearance of *P. papula* (77.60 m CCSF) that show similar stratigraphic distributions at low and mid‐latitudes (e.g., Petrizzo, 2019; Petrizzo et al., 2017; Premoli Silva & Sliter, 1995).

In general, *Lithastrinus* specimens are rare within the austral Santonian record, although *L. septenarius*, which has its HO at 99.20 m CCSF, usually occur in sufficient abundance to have a biostratigraphic significance. This utility cannot be claimed for its sister species, *Lithastrinus grilli*, whose LO is stratigraphically higher at Site U1513 and is known to be sporadic and very rare in the southern high latitudes (Watkins, 1992; Watkins & Guerra, 2020; Wise, 1983), although this datum reliably identifies the upper Coniacian in temperate regions (Blair & Watkins, 2009; Burnett, 1998; Melinte & Lamolda, 2007). *Amphizygus minimus* and *Prediscophaera desiderograndis*, whose first appearances are closely associated with the Coniacian/Santonian boundary, are well represented at Site U1513. The LO of *A. minimus,* which consistently occurs just above the Coniacian/Santonian boundary (Bergen & Sikora, 1999; Burnett, 1998; Gale et al., 2007; Hampton et al., 2007; Howe et al., 2007), is first observed at 108.64 m CCSF at Site U1513. The HO of the high‐latitude species *Thiersteinia ecclesiastica* at 101.08 m CCSF indicates direct correlation with the top of the *T. ecclesiastica* Zone, established by Wise (1983) for the Coniacian‐Santonian in the Falkland Plateau record and evident throughout the Southern Ocean (Watkins et al., 1996; Wise, 1988). Burnett (1998) placed this biohorizon in Subzone UC11b, although evidence from Site U1513 indicates a later placement in what would correlate to Subzone UC11c.

Subdivision of the middle and upper Santonian is difficult in many areas, but especially so in austral paleolatitudes. Holococcoliths, including the genus *Calculites*, are generally quite rare or absent in the Santonian and lower Campanian of the austral oceans (e.g., Watkins et al., 1996). As a result, Zone CC16 could not be differentiated at Site U1513 based on the appearance of *Calculites obscurus*. The applicability of this zonal biohorizon, though, is dubious, at best, as it is often absent from other settings (e.g., Western Interior Basin: Kita et al., 2017). In addition, *C. obscurus* has been shown conclusively to be affected by strong diachronism of its first appearance (Thibault et al., 2016). Thus, the HO of *L. septenarius* (99.20 m CCSF) is used as an alternate biohorizon for the base of Zone CC17. This zone continues to the top of the Cretaceous section at Site U1513, as no specimens of *Broinsonia parca parca*, or its ancestor *B. p. expansa* (s.s.) were observed in any sample. This age is corroborated by the presence throughout this interval and in the topmost sample at 62.70 m CCSF of *Eprolithus floralis*, which had a latest Santonian extinction.

Therefore, although the topmost sediments of the Cretaceous interval in Holes U1513A and U1513B were previously dated Santonian‐earliest Campanian by Huber et al. (2019a), re‐examination of the calcareous plankton assemblages revealed the absence of the marker taxa used to identify the base of the Campanian (see discussion in Miniati et al., 2020). Thus, the Santonian‐Campanian boundary interval is interpreted to be missing at Site U1513 and the hardground that overlies the Cretaceous sediments corresponds to a hiatus that spans from the upper Santonian to Miocene (Figure 2).

## 4 Composition of the Microfossil Assemblages

**Figure 3:**
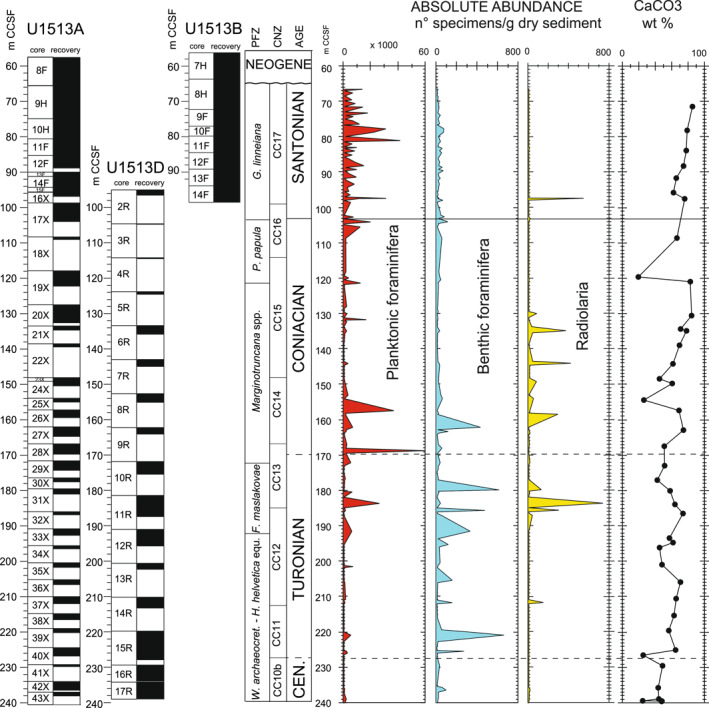
Holes U1513A, U1513B, and U1513D: absolute abundance in number of specimens per gram of dry sediments of planktonic foraminifera, benthic foraminifera, and radiolaria. CaCO_3_ content according to Huber et al. (2019a). Core recovery, age, planktonic foraminiferal and calcareous nannofossils biozonations, and abbreviations as explained in the caption of Figure 2.

Apart from calcareous nannofossils, the microfossil assemblage throughout the studied stratigraphic section at Site U1513 is composed mainly of planktonic foraminifera that reach absolute abundance values of 50,000 to 60,000 specimens per gram of dry sediment in the upper Turonian‐lower Coniacian and in the Santonian (Figure 3). Benthic foraminifera are subordinate in relative abundance and occur in high absolute abundance (600–700 specimens per gram of dry sediment) only in the Turonian to lower Coniacian sediments. Radiolaria are present from the upper Turonian to middle Coniacian and near the base of the Santonian reaching 700 specimens per gram of dry sediment. The carbonate content (CaCO_3_) parallels the calcareous microfossil absolute abundance values and shows a progressive increase from the base to the top of the studied stratigraphic interval with episodic decreases related to the presence of more clay‐rich intervals at 226.54, 196.09, 176.98, 154.32, and 119.59 m CCSF that contain a carbonate content ranging from 20 to 40wt% (Figure 3).

**Figure 4:**
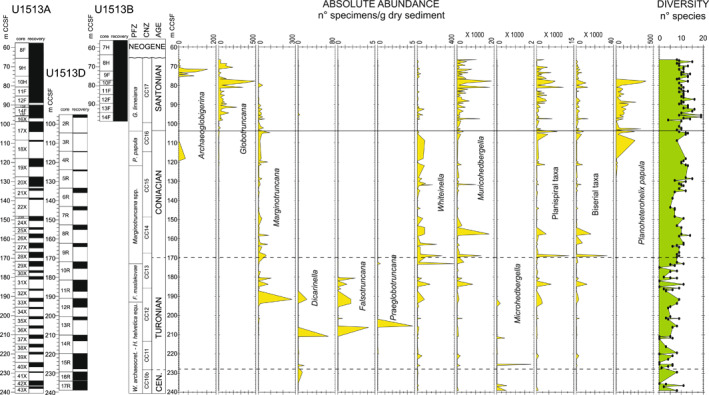
Holes U1513A, U1513B, and U1513D: absolute abundance in number of specimens per gram of dry sediments of planktonic foraminiferal genera, species and groups, and diversity of species. Core recovery, age, planktonic foraminiferal and calcareous nannofossils biozonations, and abbreviations as explained in the caption of Figure 2.

The diversity of planktonic foraminifera shows a gradual increase throughout the studied stratigraphic interval from 6–8 species in the upper Cenomanian‐Turonian to 12–18 species in the Santonian (Figure 4). The Turonian‐Coniacian interval is characterized by dominance of small‐sized specimens (125–38  μm) belonging to the genera *Muricohedbergella*, *Whiteinella*, planispiral and biserial taxa, and by low absolute abundances of the large‐sized (>125  μm) genera *Marginotruncana* and *Dicarinella*. The small‐sized *Microhedbergella* are found in the upper Cenomanian and sporadically in the Turonian (Figure 4). The increase in species richness observed across the Coniacian and Santonian intervals is coincident with the diversification of species included in *Globotruncana* and the progressive decline in abundance and extinction of all species of *Marginotruncana* which last occur in the Santonian at 79.75 m CCSF. In the same interval, the biserial and large‐sized *P. papula* shows a short stratigraphic distribution and disappears at about the same level of *Marginotruncana* (Figure 4).

**Figure 5:**
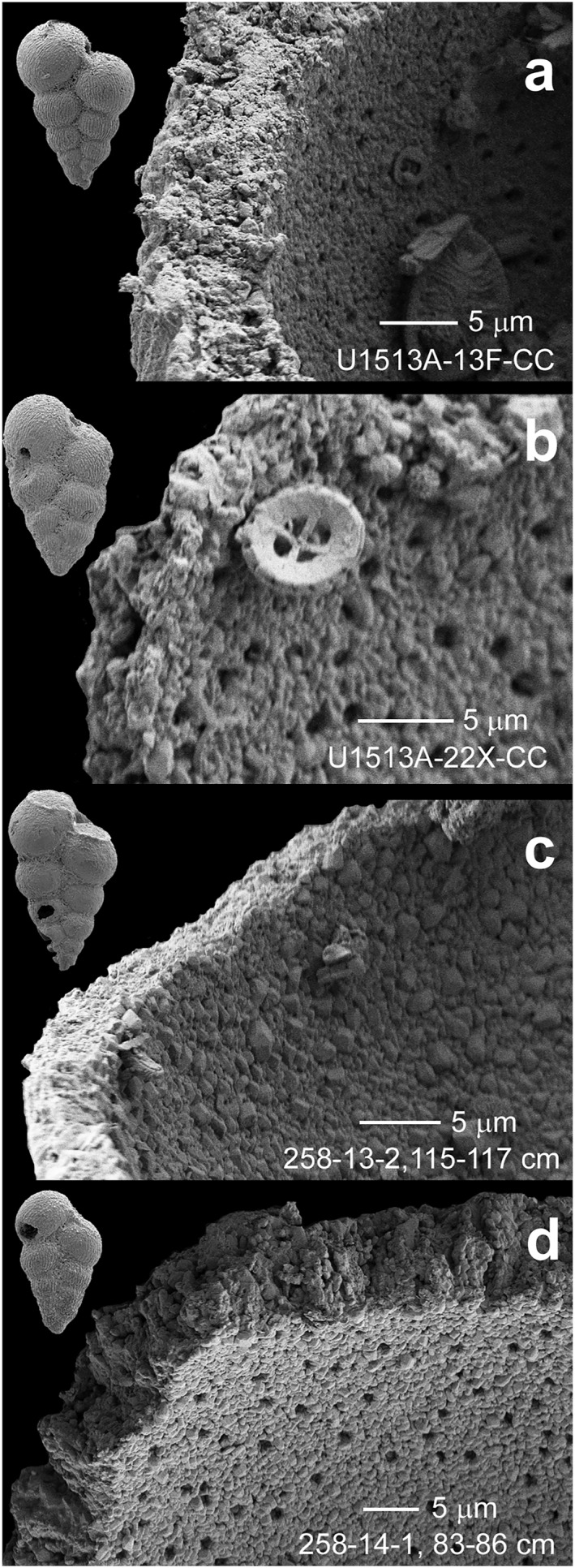
Comparison of test wall preservation among dissected specimens of *Planoheterohelix globulosa* showing interior of penultimate chamber and broken test wall surface for specimens from IODP Sites U1513 and nearby DSDP Site 258. (a) Sample U1513A‐13F‐CC, lower Santonian (*Globotruncana linneiana* Zone, Zone CC17). (b) Sample U1513A‐22X‐CC, lower Coniacian (*Marginotruncana* spp. Zone, Zone CC15). The calcareous nannofossil *Praedicosphaera columnata* (proximal view) is visible on the foraminiferal test. (c) Sample 258‐13‐2, 115–117  cm, lower Turonian (*Whiteinella baltica* Zone, Zone CC11; Huber et al., 2018). (d) Sample 258‐14‐1, 83–86  cm, upper Cenomanian (*Whiteinella baltica* Zone, Zone CC10a; Huber et al., 2018).

Preservation is good to moderate in most of the latest Cenomanian‐Santonian samples where tests exhibit slight to moderate recrystallization. Very rare specimens showing glassy preservation (=translucent tests lacking evidence of test wall recrystallization) were observed within the studied interval. Several samples in the Turonian and Coniacian intervals yield foraminifera that are strongly recrystallized and infilled with sparry calcite. Specimens showing this level of preservation were excluded from the isotopic analyses. Tests of the planktonic foraminifera *Planoheterohelix globulosa* were dissected and imaged in a scanning electron microscope (SEM) to show typical preservation of specimens analyzed for their stable isotopic composition. Test walls showing minor recrystallization (=good preservation) have wall pores that are still visible and calcite rhombs on the test interior are very small in size (Figures 5a and 5b, 5d). Test walls showing moderate recrystallization (=moderate preservation) have coarser calcite overgrowths that have infilled the original wall pores (Figure 5c).

**Figure 6:**
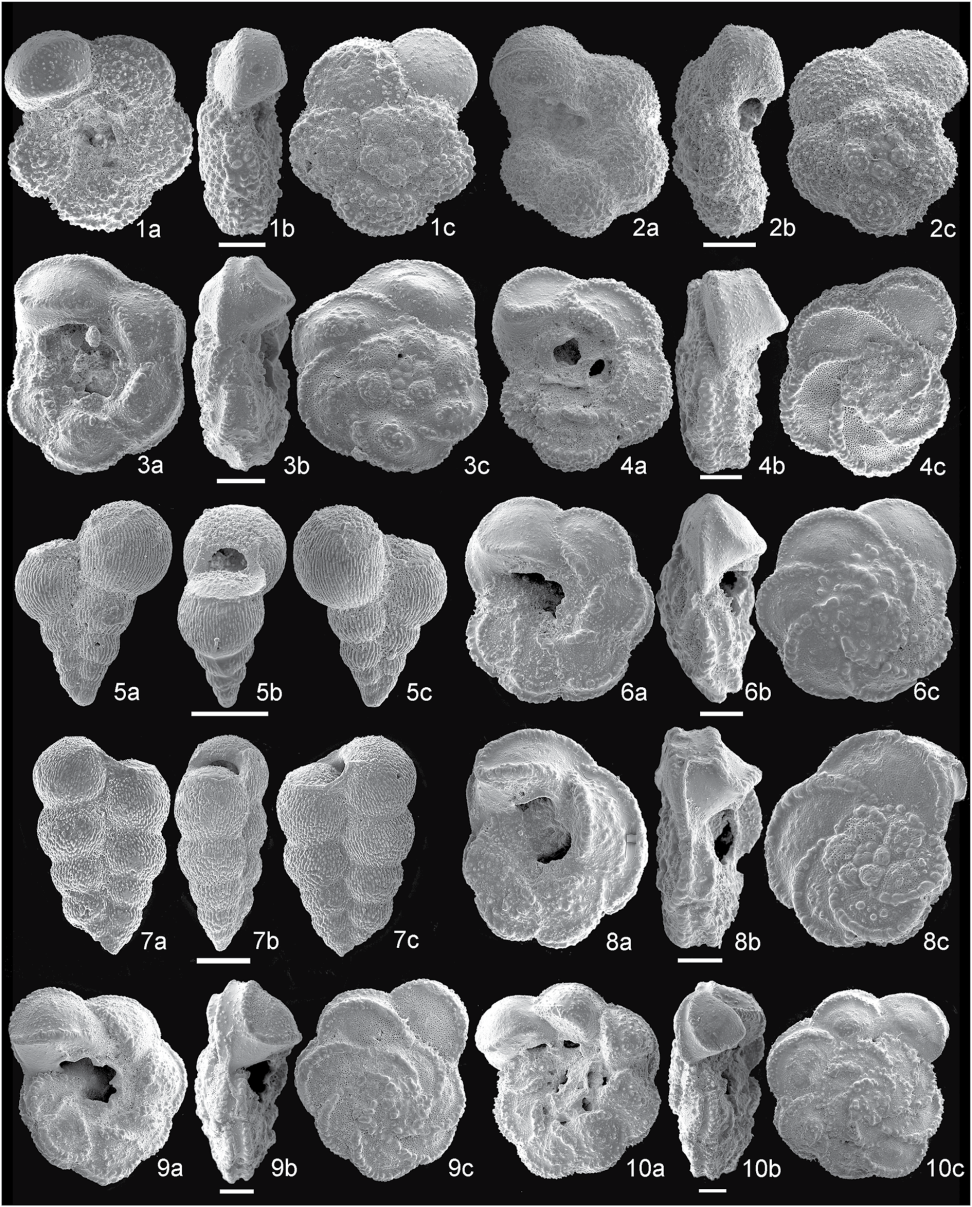
Scanning electron microscope (SEM) images of species of planktonic foraminifera. (1a–c) *Globotruncana hilli*, sample 369‐U1513B‐8H‐5, 98–100 cm. (2a–c) *Archaeoglobigerina cretacea*, sample 369‐U1513B‐8H‐6, 48–50 cm. (3a–c) *Globotruncana bulloides*, sample 369‐U1513B‐10F‐1, 50–52 cm. (4a–c) *Globotruncana linneiana*, sample 369‐U1513B‐11F‐1, 100–102 cm. (5a–c) *Planoheterohelix globulosa*, sample 369‐U1513B‐8H‐5, 98–100 cm. (6a–c) *Globotruncana arca*, sample 369‐U1513B‐13F‐2, 145–147 cm. (7a–c) *Planoheterohelix papula*, sample 369‐U1513A‐13F‐1, 52–54 cm. (8a–c) *Marginotruncana pseudolinneiana*, sample 369‐U1513B‐14F‐1, 122–124 cm. (9a–c) *Globotruncana neotricarinata*, sample 369‐U1513B‐14F‐2, 70–74 cm. (10a–c) *Globotruncana bulloides* (=*culverensis*), sample 369‐U1513B‐14F‐3, 22–24 cm.

**Figure 7:**
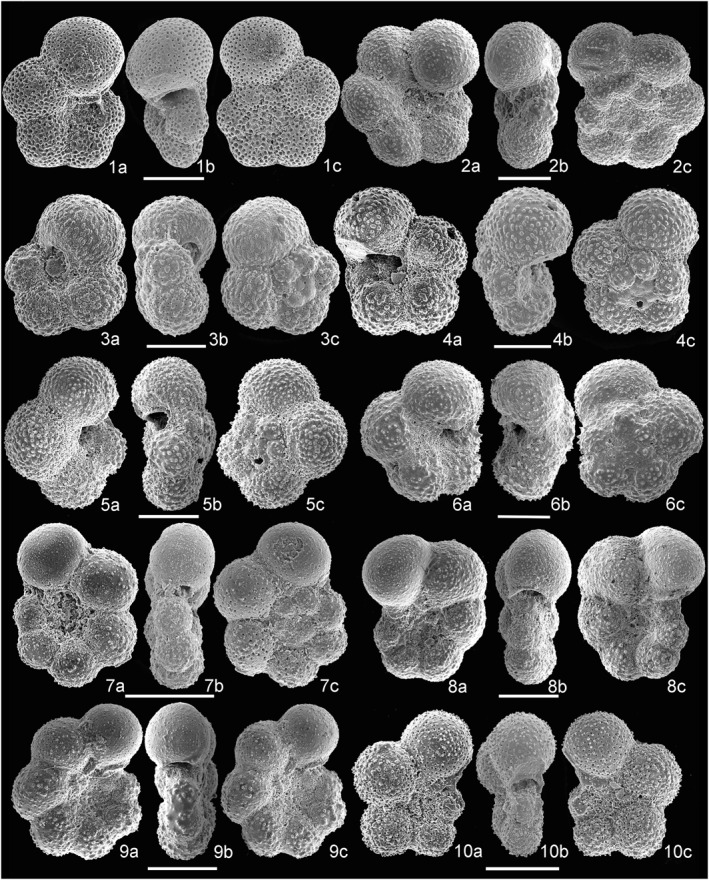
Scanning electron microscope (SEM) images of species of planktonic foraminifera. (1a–c) *Muricohedbergella crassa*, sample 369‐U1513A‐11F‐4, 60–62 cm. (2a–c) *Muricohedbergella planispira*, sample 369‐U1513B‐9F‐3, 30–32 cm. (3a–c) *Costellagerina* cf. *bulbosa*, sample 369‐U1513A‐9H‐5, 145–147 cm. (4a–c) *Costellagerina* cf. *pilula*, sample 369‐U1513A‐9H‐5, 145–147 cm. (5a–c) *Whiteinella baltica*, sample 369‐U1513B‐13F‐5, 145–147 cm. (6a–c) *Muricohedbergella delrioensis*, sample 369‐U1513B‐13F‐5, 95–97 cm. (7a–c) *Liuenella falklandica*, sample 369‐U1513A‐9H‐6, 98–100 cm. (8a–c) *Globigerinelloides alvarezi*, sample 369‐U1513B‐10F‐1, 50–52 cm. (9a–c) *Globigerinelloides ultramicrus*, sample 369‐U1513B‐11F‐1, 100–102 cm. (10a–c) *Globigerinelloides asper*, sample 369‐U1513A‐11F‐2, 143–145 cm.

**Figure 8:**
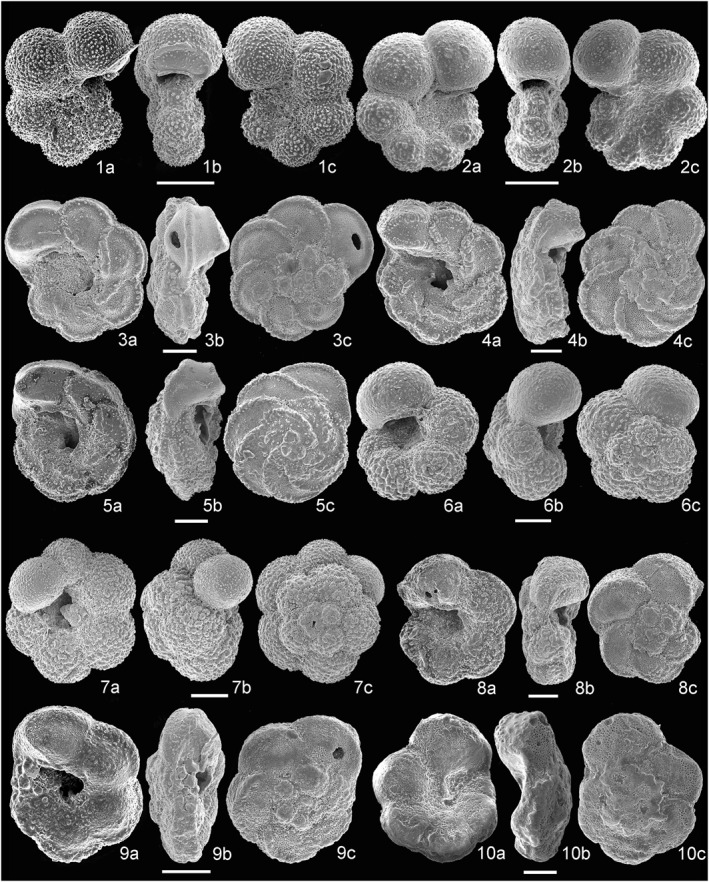
Scanning electron microscope (SEM) images of species of planktonic foraminifera. (1a–c) *Globigerinelloides prairiehillensis*, sample 369‐U1513A‐11F‐2, 143–145 cm. (2a–c) *Globigerinelloides yaucoensis*, sample 369‐U1513B‐14F‐2, 70–72 cm. (3a–c) *Marginotruncana pseudomarginata*, sample 369‐U1513A‐17X‐4, 70–73 cm. (4a–c) *Marginotruncana coronata*, sample 369‐U1513A‐17X‐4, 70–73 cm. (5a–c) *Contusotruncana morozovae*, sample 369‐U1513A‐17X‐2, 40–43 cm. (6a–c) *Whiteinella brittonensis*, sample 369‐U1513A‐24X‐1, 10–13 cm. (7a–c) *Whiteinella paradubia*, sample 369‐U1513A‐19X‐3, 75–78 cm. (8a–c) *Dicarinella marginata*, sample 369‐U1513A‐26X‐1, 88–91 cm. (9a–c) *Dicarinella hagni*, sample 369‐U1513A‐37X‐2, 111–114 cm. (10a–c) *Falsotruncana maslakovae*, sample 369‐U1513A‐33X‐1, 10–13 cm.

The stratigraphic distribution of the planktonic foramniferal species analyzed and the list of taxa occurring at Site U1513 are reported in Supporting  Information S1 and at https://doi.pangaea.de/10.1594/PANGAEA.939392. The most important species are illustrated in Figures 6, 7, 8.

Calcareous benthic foraminifera commonly occur and show good preservation in the Turonian to Santonian sediments at Site U1513. The benthic foraminiferal assemblage is characterized by the common occurrence of *Notoplanulina* spp., *Gavelinella* spp., *Anomalinoides* spp., and various gyroidinids. *Nuttallides* and *Nuttallinella* are abundant and show a continuous occurrence in mid‐Santonian sediments (91.26 and 76.50 m CCSF). Pleurostomellids occur frequently. The Polymorphinidae, the *Marginulina/Vaginulina* group, *Lenticulina* spp., and other nodosarids increase in abundance toward the top of the Cretaceous sediments. Agglutinated benthic foraminifera are present in some samples, and a slight increase in the abundance of these forms is recorded near the top of the Santonian succession (at about 75.00 m CCSF). Large gavelinellids, members of the *Notoplanulina* lineage, and *Nuttallinella* and *Nuttallides* decline in abundance up‐section where they are partially replaced in relative abundance by specimens of *Gyroidinoides* spp. In general, the Turonian‐Coniacian interval is characterized by bathyal benthic foraminiferal assemblages dominated by epifaunal taxa, whereas infaunal benthic foraminifera become more abundant toward the top of the Santonian. The list of taxa and the abundance of benthic foraminiferal groups throughout the Santonian at Site U1513 is reported in Supporting Information S1 and at https://doi.pangaea.de/10.1594/PANGAEA.939392.

## 5 Carbon Isotope Record

**Figure 9:**
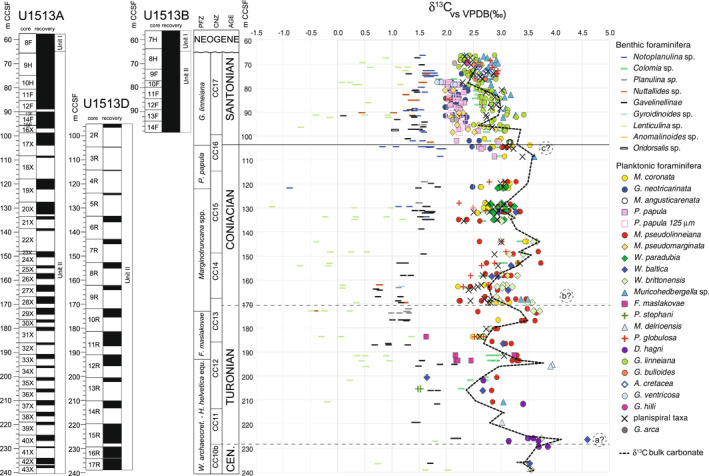
Holes U1513A, U1513B, and U1513D: δ^13^C record of bulk carbonate and benthic and planktonic foraminifera. Carbon isotope events of uncertain identification at Site U1513: a? = Cenomanian/Turonian Boundary Event, b? = Navigation Event, c? = Michel Dean Event; see Figure 1c for the carbon isotope reference curve. Core recovery, lithologic units, age, planktonic foraminiferal and calcareous nannofossils biozonations, and abbreviations as explained in the caption of Figure 2.

The carbon isotope record obtained from bulk carbonate and foraminiferal calcareous tests at Site U1513 (Figure 9; data at https://doi.pangaea.de/10.1594/PANGAEA.939392) shows generally parallel trends and consistent offsets among different foraminiferal species. These observations, combined with generally good preservation of foraminifera in the Santonian, good to moderate preservation in the Turonian‐Coniacian, and high carbonate content in all samples, support that changes in δ^13^C values record primary paleoceanographic signals. The relatively high carbon isotopic values observed at about 230 m CCSF (Figure 9) may be the local expression of the Cenomanian/Turonian Boundary Event (CTBE; Jarvis et al., 2006; Figure 1c), but high‐resolution examination of the Cenomanian‐Turonian boundary interval and OAE 2 at Site U1513 and correlation with Site U1516 (Petrizzo et al., 2021) have not been completed yet. Thus, these high δ^13^C values are not discussed further here; the OAE 2 interval at Site U1513 will be the topic of future publications.

The carbon isotope trends of both bulk carbonate and foraminiferal values observed throughout the section at Site U1513 reveal positive and negative carbon isotope excursions and inflection points, but, with a couple of exceptions, the stratigraphic patterns of peaks and troughs cannot be confidently correlated with the bulk carbonate isotopic records of the European sections (e.g., English Chalk: Jarvis et al., 2006. Bottaccione section, Italy: Sprovieri et al., 2013. Lägerdorf: Voigt et al., 2010. North Sea Basin: Eldrett et al., 2021. See also Cramer & Jarvis, 2020; Figure 1c). The record at Site U1513 does register δ^13^C minimum across the Turonian‐Coniacian boundary interval at about 162–168 m CCSF (Figure 9) consistent with the Navigation Event (Jarvis et al., 2006), which has been interpreted as the inflection point in the long‐term Late Cretaceous carbon isotope curve registered in several stratigraphic sections from Europe (e.g., Jarvis et al., 2006; Sprovieri et al., 2013; Voigt et al., 2021; Voigt & Hilbrecht, 1997) and Japan (Takashima et al., 2010, 2019). However, poor sediment recovery and poor to moderate preservation of foraminifera in several samples, especially in the Turonian‐Coniacian stratigraphic interval, hampered identification of other chemostratigraphic events. Even features observed across the Turonian‐Coniacian boundary interval, they could reflect either carbon cycling in the austral seas or be artifacts of poor recovery. Certainly, additional high‐resolution studies are needed before chemostratigraphic correlation with the European records and elsewhere can be confidently proposed for the Turonian and Coniacian.

Sediment recovery improves upward at Site U1513 and positive δ^13^C excursions in both bulk carbonate and foraminiferal data with values approaching 3.5‰ are registered across the base of the Santonian (Figure 9) and could correspond with the Michel Dean Event identified near the base of the Coniacian/Santonian boundary in the European sections (Figure 1c) including the Santonian stratotype section (Jarvis et al., 2006; Lamolda et al., 2014; Thibault et al., 2016; Voigt et al., 2010). In the Santonian interval the carbon isotope values decline by 0.5‰ and show fluctuations toward the top of the studied stratigraphic sequence (Figure 9). This trend within the Santonian is not observable in the low latitude sections (e.g., English Chalk: Jarvis et al., 2006. German chalk: Voigt et al., 2010. Italy: Sprovieri et al., 2013. Tibet: Wendler, 2013).

## 6 Foraminiferal Stable Isotope Paleoecology and Species Depth Ranking

**Figure 10:**
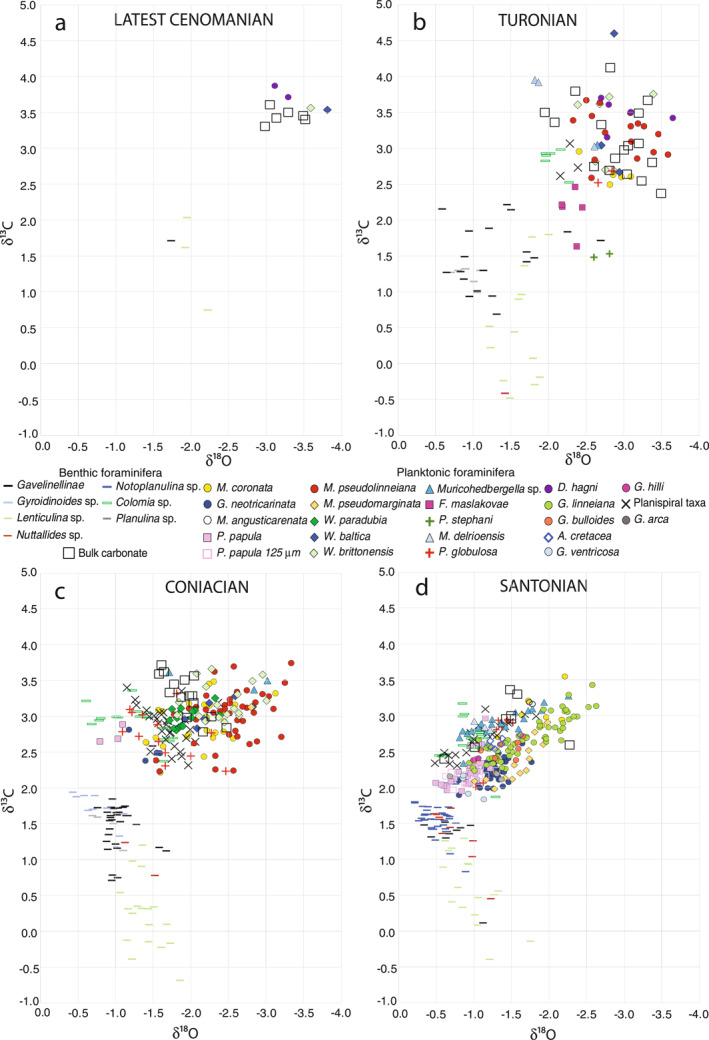
(a–d) Holes U1513A, U1513B, and U1513D: δ^18^O versus δ^13^C cross‐plots of stable isotope values measured on bulk carbonate and well‐preserved foraminiferal specimens in the latest Cenomanian (a), Turonian (b), Coniacian (c), and Santonian (d). Among planktonic foraminifera, species showing the lowest δ^18^O and the highest δ^13^C values are interpreted as surface dwellers whereas species displaying the highest δ^18^O values and the lowest δ^13^C values are interpreted as thermocline dwellers (Pearson, 1998; Pearson et al., 2001). See text for the interpretation of the depth ecologies of the taxa.

The relative ranking of carbon and oxygen isotope values measured from co‐occurring foraminiferal taxa (data at https://doi.pangaea.de/10.1594/PANGAEA.939392) permits characterization of their relative depth habitats and can be used as a proxy for determining the thermal stratification and dissolved inorganic carbon (DIC) δ^13^C gradient of the water column through time (e.g., Abramovich et al., 2003; Birch et al., 2013; D’Hondt & Arthur, 1995; Mulitza et al., 1997; Petrizzo et al., 2008; Rohling et al., 2004). Therefore, the species‐specific δ^18^O versus δ^13^C values are plotted to interpret the foraminiferal depth ecology (Figure 10). Differences in δ^18^O and δ^13^C among samples may be influenced by (a) depth migration though ontogeny; (b) peaks in abundance during different seasons for different taxa; (c) measurement errors; (d) environmental factors (e.g., pH and alkalinity); and (e) diagenetic artifacts. To minimize ontogenetic and preservational artifacts, we analyzed only the best‐preserved specimens of the most abundant species taken from a narrow size fraction.

Stable isotope analyses of bulk carbonate are also shown on the δ^18^O versus δ^13^C cross plots relative to foraminiferal data from the same time intervals (Figure 10). These measurements are mostly from calcareous nannofossils that lived in the photic zone, but they also include analyses of deeper dwelling calcareous nannofossil taxa as well as potential contributions from other calcareous biogenic constituents (e.g., benthic and planktonic foraminifera, echinoid spines).

### 6.1 Latest Cenomanian

The available data for the latest Cenomanian shown in the δ^18^O versus δ^13^C foraminiferal cross‐plot (Figure 10a) indicate a clear separation between benthic and planktonic foraminifera. *Whiteinella baltica* and *W. brittonensis* yield slightly lower δ^18^O and δ^13^C values than *Dicarinella hagni* and bulk carbonate values show a slight negative offset in δ^18^O and δ^13^C relative to the planktonic species. Although the paucity of data in this interval prevents detailed interpretations, the cross‐plots of planktonic foraminifera are consistent with their growth in a shallow depth habitat.

### 6.2 Turonian

The δ^18^O versus δ^13^C cross‐plot of foraminiferal values in the Turonian (Figure 10b) indicates differences of about 2.5‰ in both δ^13^C and δ^18^O between benthic taxa and planktonic foraminifera exhibiting the strongest surface water signal. However, there is a lack of clear separation among species within the planktonic and benthic realm along either axis. Whereas δ^13^C variations among benthic taxa could reflect variations in export productivity influencing the strength of the biological pump (MacLeod et al., 2001), with infaunal taxa migrating within the sediment column tracking food sources, it is difficult to explain why these changes might lead to the high variation among benthic δ^18^O measurements. One alternative hypothesis is that large interannual variation in circulation, perhaps on millennial timescales, within a basin strongly influenced by local processes is the source of variability observed among benthic taxa. It is noteworthy that the δ^13^C and δ^18^O values of *Colomia* are higher than co‐occurring benthic taxa because of its aragonitic test composition (Bandy, 1954; Loeblich & Tappan, 1984; Wendler et al., 2011).

Large interannual and/or seasonal variations in the surface water conditions could explain overlap among planktonic foraminiferal and the wide scatter of bulk carbonate data. Petrizzo et al. (2008) and Ando et al. (2010) recognized the influence of seasonal variations in planktonic foraminiferal data from Albian‐Cenomanian sediments recovered in western North Atlantic (Blake Nose, ODP Sites 1050 and 1052) and suggested that stable isotope separation among planktonic species reflects species proliferation during their seasonal optima. Specifically, the thin summer mixed layer is expected to experience relatively large and frequent short‐term perturbations in temperature and salinity. This difference should be reflected in greater variability in δ^18^O values in analyses of taxa that lived in the summer mixed layer than observed for analyses of taxa that lived in the thick winter mixed layer or below the seasonal thermocline (in addition to surface dwelling taxa having generally low δ^18^O values and high δ^13^C values; Petrizzo et al., 2008).

Applying this model, we propose that *W. brittonensis* and *D. hagni* in the Turonian interval (Figure 10b; Table 2) were summer mixed‐layer dwellers as interpreted for nearby Site U1516 in the Mentelle Basin (Petrizzo et al., 2020, 2021). These taxa exhibit relatively high variability in δ^18^O and δ^13^C among samples and show the lowest δ^18^O and highest δ^13^C values. *Whiteinella baltica* and *Marginotruncana pseudolinneiana* also shows relatively high variability among samples but have slightly lower δ^13^C values suggesting that they lived near the seasonal thermocline or in the thick winter mixed layer. *Marginotruncana coronata*, *P. globulosa*, and planispiral taxa have isotopic values with relatively little variation among samples and are hypothesized to have lived below the seasonal thermocline in agreement with previous data from other southern high latitudes sites (Falkland Plateau Site 511, Mentelle Basin Site 258 and Site U1516; Huber et al., 1995, 2018; Petrizzo et al., 2020; Figure 1a). Low δ^13^C values are typical for Cretaceous biserial taxa (*P. globulosa*; Huber et al., 1995, 1999; MacLeod et al., 2000, 2001; 2005) and may reflect stronger disequilibrium fractionation due to their presumed faster growth rate, which has been inferred for opportunistic groups (e.g., Bornemann & Norris, 2007; Hart, 1980; Leckie, 1987; Premoli Silva & Sliter, 1999). The stable isotope record of *F. maslakovae* and *Praeglobotruncana stephani* is consistent with previous interpretations (Mentelle Basin Site U1516: Petrizzo et al., 2020; Falkland Plateau Site 511: Huber et al., 1995; Tanzania: Wendler et al., 2013) that show relatively little variability between samples, with the highest δ^18^O values and usually the lowest δ^13^C values, suggesting that they probably lived below the seasonal thermocline, possibly near the permanent thermocline. The depth ecology of *Muricohedbergella*’s species is more difficult to interpret because of the variability in δ^18^O and δ^13^C values among samples, although its variable δ^13^C values could indicate seasonal variations in the surface waters.

**Table 2:** Depth Ecology of the Planktonic Foraminifera Species Occurring at Site U1513 According to Their Species‐Specific δ^18^O Versus δ^13^C Values

**Mixed layer**	**Thick mixed layer or seasonal thermocline**	**Permanent thermocline**
**Surface dwellers**	**Intermediate dwellers**	**Thermocline dwellers**
TURONIAN		
*Whiteinella brittonensis*	*Marginotruncana pseudolinneiana*	*Marginotruncana coronata*
*Dicarinella hagni*	*Whiteinella baltica*	*Falsotruncana maslakovae*
	*Muricohedbergella*	*Praeglobotruncana stephani*
		Biserial taxa
		Planispiral taxa
		
CONIACIAN		
*Muricohedbergella*	*Whiteinella baltica*	*Globotruncana neotricarinata*
*Marginotruncana pseudolinneiana*	*Whiteinella paradubia*	*Planoheterohelix papula*
*Whiteinella brittonensis*	*Marginotruncana coronata*	Biserial taxa
		Planispiral taxa
		
SANTONIAN		
*Muricohedbergella*	*Archaeoglobigerina cretacea*	*Globotruncana neotricarinata*
*Marginotruncana coronata*	*Globotruncana bulloides*	*Planoheterohelix papula*
*Globotruncana linneiana*		*Globotruncana hilli*
*Marginotruncana angusticarenata*		*Globotruncana arca*
		*Globotruncana ventricosa*
		*Marginotruncana pseudomarginata*
		Biserial taxa
		Planispiral taxa

*Note*. See text for further explanation.

### 6.3 Coniacian

In the Coniacian, the δ^18^O versus δ^13^C cross plot (Figure 10c) indicates differences of about 2.0‰ between epibenthic foraminifera and planktonic foraminiferal species with the latter exhibiting the strongest surface water signal. In both δ^13^C and δ^18^O values there is good separation between planktonic and benthic values. In addition, benthic δ^18^O values span a much smaller range and the degree of correlation between δ^18^O and δ^13^C values of planktonic foraminiferal species is higher compared to the Turonian data. These changes suggest increased stratification of the water column and lower interannual variation in sea floor waters. However, planktonic foraminiferal data still show moderate overlap among species that could indicate large interannual and/or seasonal variations in the surface water conditions.

*Muricohedbergella* show low δ^18^O and high δ^13^C values and are interpreted as summer, mixed‐layer dwellers (Figure 10c; Table 2). *Marginotruncana pseudolinneiana* shows the lowest δ^18^O and the highest δ^13^C values and is interpreted as summer mixed‐layer dweller. It also has high intra‐specific variability among samples consistent with relatively large and frequent short‐term perturbations in temperature and nutrient concentrations. Similarly, *M. coronata* exhibits relatively high variability in δ^18^O and δ^13^C among samples suggesting that it lived in the thick winter mixed layer, and it may also have lived in deeper ecological niches during its ontogeny or had the potential to expand its numbers early in the spring before significant warming and ^12^C depletion of surface water DIC had occurred. *Whiteinella baltica* and *Whiteinella paradubia*, show a slightly higher δ^18^O and lower δ^13^C values indicating a depth habitat near the seasonal thermocline or in the winter mixed layer, or population blooms early in the season before surface waters are depleted in ^12^C. *Whiteinella brittonensis,* in contrast, shows a similar degree of variability but higher δ^13^C and lower δ^18^O values suggesting its preference for growth during the summer season. Planispiral and biserial (*P. globulosa*) taxa register small δ^18^O and high δ^13^C intra‐specific variability among samples indicating a depth habitat below the seasonal thermocline or near the permanent thermocline. A habitat in the permanent thermocline is inferred for *G. neotricarinata,* which yields the highest δ^18^O and nearly the lowest δ^13^C values and for *P. papula* that exhibits the highest δ^18^O and relatively low δ^13^C values. Bulk carbonate cross‐plots are intermediate between those of the upper surface dwelling and thermocline dwelling planktonic species.

### 6.4 Santonian

**Figure 11:**
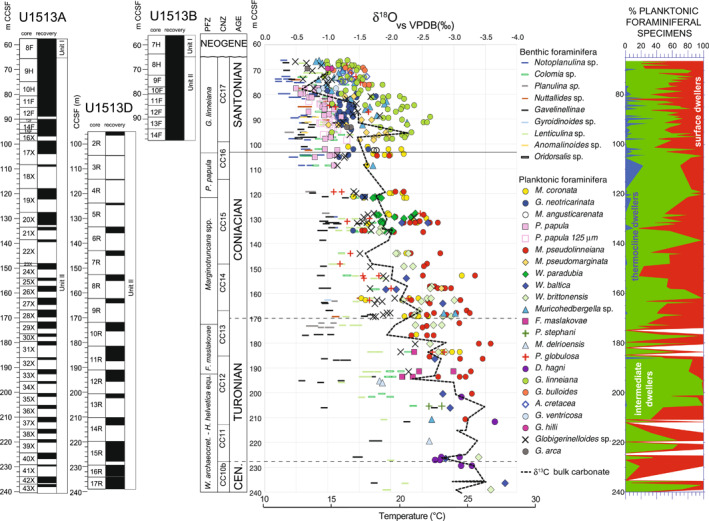
Holes U1513A, U1513B, and U1513D: δ^18^O record of bulk carbonate and foraminifera compared with the percent distribution of thermocline (blue), intermediate (green), and surface dweller (red) planktonic foraminifera; barren samples are shown in white. Core recovery, lithologic units, age, planktonic foraminiferal and calcareous nannofossils biozonations, and abbreviations as explained in the caption of Figure 2.

**Figure 12:**
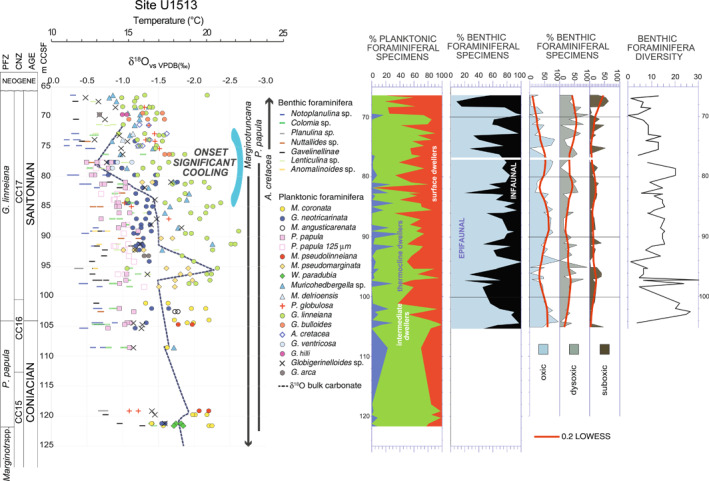
The upper Coniacian‐Santonian interval at Site U1513: δ^18^O record of bulk carbonate and foraminifera. Percent abundance of thermocline (blue), intermediate (green), and surface dweller (red) planktonic foraminifera. Percent abundance of epifaunal (light blue), infaunal (black), oxic, dysoxic, suboxic benthic foraminifera, and taxa diversity. The stratigraphic distribution of *Marginotruncana*, *Archaeoglobigerina cretacea*, and *Planoheterohelix papula* is shown. The onset of the significant cooling in the Santonian is placed at the abrupt decline in the δ^18^O values of the surface dweller planktonic foraminifera. Age, planktonic foraminiferal and calcareous nannofossils biozonations, and abbreviations as explained in the caption of Figure 2. See text for further explanation.

The depth ecology of Santonian foraminifera is consistent with previous stable isotopic interpretations from Site U1513 (Petrizzo et al., 2020) that reveal they lived in a well stratified water column. There is a clear separation in isotopic values between planktonic and benthic species as well as good separation among taxa living within the surface waters (Figure 10d; Table 2). Differences between planktonic foraminifera and epibenthic foraminifera remain about 2.0‰ in both δ^13^C and δ^18^O, similar to the Coniacian data. Planktonic foraminifera exhibit high inter‐specific variability in δ^18^O and δ^13^C values, but the overlap in fields occupied by different taxa is reduced relative to earlier time slices and correlation between δ^18^O versus δ^13^C is increased (Figure 10d). Separation among Santonian taxa is arguably even more apparent in the stratigraphic plots (Figures 9, 11, and 12) than the cross‐plots (Figure 10d) as declining trends in both isotopic measurements for all taxa through time are masked when the data are all plotted on the same isotopic axes. Regardless of the visualization scheme, these data indicate well‐developed stratification, with a stable and relatively well‐developed thermocline distinct from the surface mixed layer.

*Marginotruncana coronata* shows the lowest δ^18^O and the highest δ^13^C values suggesting this species was a summer mixed‐layer dweller at high latitudes during the Santonian. *Globotruncana linneiana* and *Muricohedbergella* exhibit very high variability in δ^18^O and δ^13^C of more than 1‰ among samples indicating they had peak population numbers spanning two or more seasons or were changing depth habitats within the mixed layer probably because of variations in temperatures and nutrients supplies. *Archaeoglobigerina cretacea* and *Globotruncana bulloides*, show a low isotopic variability that suggests they lived in the thick winter mixed layer. *Planoheterohelix globulosa* and planispiral taxa exhibit isotopic values indicating a depth habitat below the seasonal thermocline. The highest δ^18^O values and lowest δ^13^C values of *P. papula*, *G. neotricarinata*, *G. hilli*, *Globotruncana arca*, *Globotruncana ventricosa*, and *Marginotruncana pseudomarginata* suggest a depth habitat within or below the thermocline. The bulk carbonate cross‐plots are closer to those of surface mixed layer species than thermocline species.

### 6.5 Additional Remarks

Especially evident in the present planktonic foraminiferal data set is the depth distribution of *M. coronata*, which shows an isotopic signature indicative of a relatively deep and cold habitat in the Turonian (Figure 10b). Then, in the Coniacian (Figure 10c), isotopic values indicate *M. coronata* increasingly lived in water with a summer surface water signature suggesting it migrated toward sharing the ecological niches in the mixed layer occupied by *M. pseudolinneiana*. In the Santonian (Figure 10d), immediately before the time of its extinction, its isotopic values overlap with the surface‐dwelling *G. linneiana*.

The low isotopic values with large scatter in δ^13^C of *Lenticulina* in all time intervals are consistent with its inferred epifaunal to infaunal habitat and a strong vital effect, possibly related to a fast metabolism, reproduction and calcification (Wendler et al., 2013). Large epifaunal benthic foraminifera, gavelinellids, and *Notoplanulina* sp. show similar δ^13^C and δ^18^O values, and the values of the gavelinellids fall in the range of the *Notoplanulina* sp. that exhibit a slightly broader variability in the δ^13^C values.

## 7 Paleotemperature Estimates

Late Cenomanian‐Santonian foraminifera from Site U1513 yield oxygen isotope ratios that suggest warmest temperatures during the latest Cenomanian‐early Turonian, followed by a plateau of warm temperatures in the Turonian‐early Coniacian before a progressive decline throughout the Coniacian, reaching coolest temperatures in the interval studied within the Santonian (Figure 11). These data nicely mirror previous paleotemperature estimates (Figure 1c; see for instance Friedrich et al., 2012; Huber et al., 2018; O’Brien et al., 2017; and references herein).

In the uppermost Cenomanian sediments (239.06–229.47 m CCSF), δ^18^O for planktonic foraminifera are notable for their remarkably low values of ‒3.8‰ that suggest paleotemperatures of 27°C in the surface waters. In the same stratigraphic interval, epifaunal benthic foraminiferal δ^18^O values range from −1.7 to −2.3‰ (18°C–20°C).

Throughout the Turonian (229.47–172.10 m CCSF), apparent temperatures remain quite high with the lowest δ^18^O values consistently falling between −3.0‰ and −3.5‰. The range of planktonic foraminifera δ^18^O values in the Turonian varies from −1.8‰ to −3.7‰ (18°C–27°C). Benthic foraminifera δ^18^O values are between −0.5‰ and −2.6‰ and seem to indicate a high variability (12°C–22°C) which is difficult to interpret. Variation could be related to changes in the pattern of the deep‐water circulation in a basin influenced by local processes complicated by factors such as depth in the sediment taxa live and variations in carbonate ion concentrations especially in pore waters.

There is a long‐term gradual increase in planktonic foraminifera δ^18^O values by 0.5‰–1.0‰ through the Coniacian (172.10–104.45 m CCSF). The lowest measured value in lower Coniacian is −3.3‰ (25°C) at 153.00 m CCSF and values increase to −2.2‰ (20°C) in the youngest Coniacian samples. The upward increase of the mean foraminiferal δ^18^O values is confirmed by the similar trend of the bulk carbonate data. Over the same interval, maximum planktonic δ^18^O values increase from −1.7‰ to −0.7‰. Benthic foraminifera δ^18^O values are between −0.6‰ and −1.8‰ and are largely unchanged through most of the Coniacian (172.10–119.16 m CCSF); they increase by 0.5‰ in the upper 10 m of the Stage. The difference in trends between planktonic and benthic taxa results in a progressive decrease of the surface to seafloor δ^18^O gradient suggesting a decreased vertical temperature gradient through time.

Oxygen isotope ratios for both planktonic and benthic foraminifera increase to higher values in the Santonian (Figure 11). Sea surface paleotemperatures estimated from the shallow dwelling planktonic foraminifera shift from 20°C near the base of the Santonian at 104 m CCSF to 23°C at 90 m CCSF before decreasing to 14°C at the top of the studied interval (66.40 m CCSF). Much of this change occurs between 85.38 and 72.79 m CCSF (Figures 11 and 12). As in the Coniacian, bulk carbonate δ^18^O values parallel those of surface water, planktonic foraminiferal trends. The highest δ^18^O values of planktonic taxa indicate cooler temperatures varying only slightly from 15°C to 12°C in the thermocline during the Santonian, and benthic temperatures are estimated to have fallen only slightly from 12°C to 10°C across the interval. All together these values suggest continued cooling during the Santonian that accelerated during the middle portion of the Stage and affected the upper part of the water column more strongly than the deeper surface waters and the seafloor.

Comparison of the planktonic and benthic foraminiferal δ^18^O record and the paleotemperature estimates at Site U1513 with the data from DSDP Site 258 (Huber et al., 2018) which is located 1.1  km west‐northwest from Site U1513, show a good correspondence of values between the two sites in the interval from the earliest Cenomanian to the Coniacian. In the Santonian the low recovery of sediments and low diversity of the calcareous plankton assemblages at Site 258 (Herb, 1974; Huber et al., 2018) prevent the observation of the onset of the cooling in both surface waters and seafloor as documented at Site U1513.

## 8 Paleoceanography and Onset of the Long‐Term Cooling in the Santonian

### 8.1 Planktonic Foraminiferal Patterns

The patterns of the planktonic foraminiferal assemblages in terms of diversity, depth habitats and paleotemperatures described above and illustrated in Figures 9, 10, 11 reveal some differences throughout the studied stratigraphic interval that are used to interpret variations in the vertical structure of the water column. The Cenomanian‐Turonian boundary interval is characterized by high sea surface paleotemperatures of about 27°C (Figure 11), by the dominance of surface‐dwelling taxa that alternate with taxa dwelling at intermediate water depths, and by an apparent absence of obligate thermocline dwellers (Figure 11). Moreover, typical Tethyan species are absent at Site U1513, including the zonal marker and intermediate dweller *H. helvetica* that is commonly recorded from low latitudes to as far south as 50°S at the Kerguelen Plateau (Petrizzo et al., 2020). These observations together with the paleotemperature values inferred from δ^18^O foraminiferal data, might indicate a reduced vertical temperature gradient between mixed layer and thermocline waters. The occurrence of common microhedbergellids (Figure 4), interpreted as opportunistic species because of their small size and high reproductive potential, further suggests a paleoceanographic regime affected by episodes of enhanced eutrophy, in agreement with data from across the Cenomanian‐Turonian boundary interval at Site U1516 which is located only few kilometers distant from Site U1513 (Figure 1b; Petrizzo et al., 2021).

Surface dwelling taxa continue to dominate in the lower Turonian (Figure 11) until they are replaced in abundance by the intermediate and thermocline depth dwellers *Marginotruncana* and planispiral taxa that together with the thermocline dwellers *Falsotruncana* and *Praeglobotruncana* comprise the mid‐to late Turonian assemblages (Figure 4). The fluctuation in abundance of the surface, intermediate, and thermocline dwellers observed in the Turonian and Coniacian point to a relatively stable paleoceanographic setting with a thermal gradient in the surface waters. In the Coniacian‐Santonian interval, fluctuations in abundance of the trophic groups (Figure 11) are associated with the progressive decrease of paleotemperatures in the surface water as recorded by the bulk carbonate and foraminiferal δ^18^O values.

The distribution of the trophic groups at Site U1513 during the Turonian‐Santonian is comparable with the foraminiferal record reported from the Northeast Georgia Rise and the Kerguelen Plateau (Petrizzo et al., 2020), located in the southern Indian Ocean at 58°S and 50°S of paleolatitude, respectively (Figure 1a). The composition of the assemblages also reflects the presence of a well‐defined thermocline or a thick mixed layer suitable to accommodate a large number of ecological niches. In addition, the presence of Tethyan species at Site U1513, albeit only in low abundance, is consistent with the Mentelle Basin (about 60°S of paleolatitudes) being just south of the Tethyan Bioprovince. This Bioprovince reached its maximum poleward expansion during the Turonian‐early Santonian hot greenhouse climate (Petrizzo et al., 2020).

The Tethyan affinity of the Turonian‐Santonian planktonic foraminifera at Site U1513 is also in agreement with the presence of a subtropical gyre inferred to pass along the southern edge of India and eastward to the northwestern edge of Australia, and of a subantarctic gyre inferred to flow northward along Antarctica and then eastward into the southern Indian Ocean (Figure 1a; Huber, 1992; Pucéat et al., 2005). North‐south migration of the boundary between these two gyres flowing near Site U1513 may explain the fluctuations in abundance and composition of the three trophic groups responding to the variation in the relative local importance of either the warmer, northern or colder, southern gyre (Figure 11).

The topmost sedimentary sequence in the Santonian contains planktonic foraminiferal assemblages characterized by the loss of most Tethyan thermocline species. This change in composition of the assemblages is similar to the record previously observed at the Northeast Georgia Rise and Kerguelen Plateau (Figure 1a). At these localities the changes in the foraminiferal population dynamics have been interpreted to coincide with the development of the Transitional Bioprovince at mid‐high latitudes and to the onset of the surface water cooling in the latest Santonian (Petrizzo et al., 2020). The onset of cooling in the Santonian was also documented at mid‐latitudes in the Exmouth Plateau (Indian Ocean, NW Australia; 47°S of paleolatitudes, Figure 1a) by observed changes in species composition of the planktonic foraminiferal assemblages and foraminiferal stable isotope values (Falzoni et al., 2016; Petrizzo, 2002; Petrizzo et al., 2020).

In terms of composition of the planktonic foraminiferal assemblages, the interval of relatively rapid cooling (Figure 12) includes (a) the extinctions of *P. papula* at 77.60 m CCSF and all species of the genus *Marginotruncana* (79.75 m CCSF), (b) the appearance of *A. cretacea* (75.29 m CCSF), and (c) an increase in abundance of *G. bulloides*. The extinction of *Marginotruncana* in the Santonian might be explained by the stress of cooling combined with likely competition exerted by species of the diversifying *Globotruncana* lineage which occupied similar depth habitats as species of *Marginotruncana*, a possibility previously postulated by Falzoni et al. (2018).

The composition of the planktonic foraminiferal assemblages and the isotopic record at Site U1513 clearly document the first representatives of species within the genus *Globotruncana* (*G. neotricarinata*, *G. ventricosa*, and *G. arca*) in the Coniacian, which represent taxa that diverged from their keeled shallow‐dwelling marginotruncanid ancestors and occupied a deeper/colder habitat (Figures 10, 11, 12). Subsequently, *Globotruncana*’s species migrated upward in the water column as their ancestors disappeared and surface‐ocean temperatures decreased. An exception is *G. linneiana* that inhabited the same shallow ecological niches of its ancestor *M. pseudolinneiana* (Figures 10, 11, 12). Therefore, the onset of significant cooling during the Santonian fostered the expansion of the deep/cold ecological niches that favored the proliferation in number of species of the globotruncanids (including *A. cretacea*). These species started a major phase of diversification in this time interval (e.g., Caron & Homewood, 1983; Hart, 1999; Petrizzo, 2000; Premoli Silva & Sliter, 1999), and negatively affected all shallow‐dwelling taxa that had evolved during hotter greenhouse times (Turonian‐Coniacian) such as species of *Marginotruncana*. In this paleoceanographic context, the extinction of the biserial and deeper *P. papula* could be ascribed to competition with the evolving globotruncanids in the cooler ecological niches.

### 8.2 Benthic Foraminiferal Patterns in the Santonian

The paleoceanographic signal of foraminiferal δ^18^O and δ^13^C values in the interval recording the cooling is also reflected by changes in the composition of the benthic foraminiferal assemblages (Figure 12). In general, throughout the Santonian, benthic foraminiferal specimens decrease in absolute abundance and in diversity with values for species richness decreasing from 25 species to 10 species at the top of the stratigraphic sequence (Figures 3 and 12). At the Coniacian‐Santonian transition, epifaunal and oxic taxa dominate the assemblages, whereas throughout the Santonian to 92.30 m CCSF, a significant decline in oxic benthic foraminifera is observed balanced by the marked relative increase of infaunal dysoxic taxa and a slight increase of suboxic taxa. This shift is followed by the occurrence of a more stable foraminiferal assemblage dominated by epifaunal, oxic taxa until 76.50 m CCSF (Figure 12). The overlying stratigraphic interval registers an increase in agglutinated and opportunist benthic taxa that are tolerant of oxygen undersaturated environments (e.g., marginulinids, gyroidinids, and polymorphinids), together with a progressive decline of epifaunal, oxic benthic foraminifera (Figure 12).

A combination of environmental factors might have influenced the benthic foraminiferal communities including temperature, paleocurrents, and associated changes in their behavior and nature, as well as changes in paleodepth and ocean chemistry. The proximity of Site U1513 to the west Australian continental margin and the possible terrigenous input is a factor that could significantly influence the distribution of the benthic foraminifera. However, the long‐term record of benthic foraminifera across the Santonian interval does not strictly indicate episodes of increased continental weathering that could trigger low oxygen conditions for short periods.

A possible explanation for the stepwise decline of the benthic foraminiferal abundance and diversity toward the top of the Santonian (Figures 3 and 12), and the gradual shifts in the assemblage composition from abundant oxic taxa to more dysoxic and suboxic taxa in the interval across the onset of cooling (Figure 12), could be changes in water circulation. The configuration of a tropical to subtropical gyre system that facilitated the Tethyan influence during the Turonian‐Santonian interval (Huber, 1992; Petrizzo et al., 2020; Pucéat et al., 2005) and the convergence of two oceanic gyres in the surface water (Figure 1a) might have had a decisive influence on the bottom water and, thus, on the distribution of epifaunal oxic benthic taxa at Site U1513. A possible gradual change from the influence of Tethyan paleocurrents to a more pronounced austral influence, would have resulted in cooler waters at the sea floor, a possibility consistent with a shift from older to younger water masses carrying less oxygen (Southern Component water: e.g., Donnadieu et al., 2016; Ladant et al., 2020; Moiroud et al., 2016; Pucéat et al., 2005; Robinson et al., 2010).

### 8.3 Remarks on the Onset of Cooling

The oxygen isotope data from Site U1513 provide a detailed record of the timing and pattern of the cooling phase in the Southern Hemisphere at about 60°S indicating that cooling began by the start of the Coniacian (Figure 11) in agreement with many paleotemperature compilations (e.g., Ando et al., 2013; Falzoni et al., 2016; Friedrich et al., 2012; Huber et al., 2002, 2018; Li & Keller, 1999; Linnert et al., 2014; O’Brien et al., 2017; Scotese et al., 2021; Figure 1c). Change was gradual over most of this interval at Site U1513, but a relatively brief interval of pronounced cooling marking a potential tipping point in the climatic transition from the Late Cretaceous hot to coolhouse occurred in the Santonian. This cooling step coincides with a significant increase in the Cretaceous latitudinal paleotemperature gradient.

The δ^18^O values of benthic and planktonic foraminifera show convergence near the top of the Santonian. Specifically, at Site U1513 in the interval from 85.38 to 72.79 m CCSF (Figures 11 and 12), the onset of significant cooling in the water column is nicely registered by the isotopic record of benthic and planktonic foraminifera, which indicates a decrease in temperature of about 6°C and 3°C in surface and sea floor waters, respectively. Specifically, data indicate a mean paleotemperature decrease by about 2°C–4°C in the entire water column that is more pronounced in the shallower part of the surface waters (Figures 11 and 12).

A small vertical δ^18^O and δ^13^C gradient is observable in this interval as the extremes of benthic and planktonic foraminifera values and the wide range of values in both isotope systems are reduced compared to the interval below (Figures 9 and 11). Moreover, the overlap of values of deeper planktonic (*P. papula*, *G. neotricarinata*, and planispiral taxa) and benthic foraminifera indicates a small separation between the lower sea surface and the seafloor waters (Figures 9, 11, and 12). In the same stratigraphic interval, the summer mixed layer dwellers *G. linneiana* and *Muricohedbergella* show an increase of 1.0‰ of the δ^18^O values and the thermocline *P. papula* and the benthic foraminifera shift to higher values by 0.5‰. This observation points to increasing mixing in the water column with loss of the stratified habitats of many planktonic foraminiferal taxa and reduction of the bioproduction in benthic foraminifera which declined in abundance.

The record of a significant decrease of the sea surface temperatures by 2°C in the late Santonian was also derived from TEX_86_ data obtained from sediments of the Demerara Rise in the western equatorial Atlantic Ocean (Forster et al., 2007) at 2°N–3°N of paleolatitude (Sagumana & Ogg, 2006). Additionally, a decrease in paleotemperature of about 2°C–3°C was observed in the bulk carbonate oxygen isotopes record of the late Santonian sediments from the English Chalk (Southern England) in the pioneering work by Jenkyns et al. (1994). A cooling trend was also shown for the late Santonian to early Campanian Mooreville Chalk sequence in the northeastern Gulf of Mexico (Liu, 2009). Conversely, the climatic transition from the hot to the coolhouse is placed within the first 3–4 Myr of the Campanian by Ando et al. (2013) based on the isotope record of benthic foraminifera from Shatsky Rise (IODP Site U1348, NW Pacific) located near the equator in the Late Cretaceous (Sager et al., 2005). A similar observation that infers the onset of cooling in the early Campanian is reported from Falkland Plateau (DSDP Site 511: paleolatitude about 61°S–56°S; Figure 1a) based on the foraminiferal isotopic record (Huber et al., 2018). Therefore, the beginning of the significant cooling seems to be diachronous among localities, even at similar paleolatitudes. Potential reasons for this are true diachroneity or imprecision in dating related to the different age models used in each study.

The changes in composition of the foraminiferal assemblages at Site U1513 might indicate that the onset of the cooling was more pronounced at this site than in lower latitudes. For instance, the extinction of the genus *Marginotruncana* at Site U1513 and at the Exmouth Plateau (Petrizzo, 2000) is recorded in the Santonian, thus, at a time earlier compared to the tropical Tethyan record that document the faunal turnover of the keeled taxa in the early Campanian (e.g., Coccioni & Premoli Silva, 2015; Robaszynski & Caron, 1995). This observation is not surprising considering the high latitude amplification of responses to greenhouse forcing.

## 9 Conclusions

Site U1513 in the Mentelle Basin recovered a latest Cenomanian to Santonian sedimentary sequence deposited at about 60°S in the Late Cretaceous (Hay et al., 1999; Müller et al., 2016; Scotese, 2016; van Hinsbergen et al., 2015). Biostratigraphic results based on planktonic foraminifera and calcareous nannofossils reveal a Tethyan affinity for some of the assemblages and confirms the reliability of the mid‐to high latitude biozonation for planktonic foraminifera (Petrizzo et al., 2020) except in the latest Cenomanian‐early Turonian when Tethyan marker species were absent at Site U1513.

The carbon isotope record at Site U1513 shows a positive excursion correlated with the plateau of high carbon isotopic values documented across the Cenomanian‐Turonian boundary interval in stratigraphic sections located at low (e.g., English Chalk: Jarvis et al., 2006) and high latitudes (Site U1516: Petrizzo et al., 2021). However, the carbon isotope curve from the late Turonian to Santonian at Site U1513 is difficult to interpret and to correlate across latitudes because of its low resolution due to low sediment recovery in the Coniacian interval, and the very different paleoceanographic setting of Mentelle Basin compared to the European chalk sea and other low latitude sedimentary basins.

Carbon and oxygen isotope values measured on planktonic foraminiferal taxa provide good constraints on the paleoecological preferences and depth habitats of several species. Ecological information for some species is reported for the first time in this study. The isotopic data allow interpretation of the evolution of surface water stratification through the Late Cretaceous and show fluctuations in relative thickness of the mixed layer and thermocline. Particularly interesting is documentation that *M. coronata*’s preferred habitat migrated from deeper waters in the Turonian to shallower water in the Santonian.

Paleotemperature estimates based on the δ^18^O ratios of foraminiferal tests nicely document warmest temperature of the surface (27°C) and bottom water (20°C) in the latest Cenomanian‐early Turonian, that progressively decrease thought time and reach minimum values of 14°C and 11°C at the surface and sea floor, respectively, in the Santonian.

Finally, the foraminiferal and stable isotope data presented in this paper reveal that the climatic transition from the Cretaceous hot to the coolhouse was not smoothly gradual. Cooling began by the beginning of the Coniacian and was gradual during the Stage but exhibits an accelerated step within the Santonian. The Santonian cooling event affected both surface and bottom water masses according to changes in the composition and abundance of the foraminiferal assemblages, although the cooling was more dramatic in the surface waters than at the sea floor.

Planktonic foraminifera underwent extinctions of both deep (*P. papula*) and surface (*Marginotruncana*) dwellers during the Santonian indicating a loss of ecological niches resulting from reduction of the vertical thermal gradient and of possible increased mixing within the upper water column. Benthic foraminifera document a transition from oxic to dysoxic conditions at the seafloor, and an increase in opportunistic taxa and decrease in diversity reflecting a decline in oxygenation and organic flux at the seafloor. The convergence of the subtropical and subantarctic gyres nearby Site U1513 could explain the paleotemperatures and the foraminiferal community changes registered in the surface and bottom water. At a wider paleoceanographic scale, the results of this study corroborate the hypothesis that the onset in the Santonian of the Late Cretaceous long‐term cooling was caused by changes in oceanic circulation and enhanced meridional exchanges presumably coupled with decreased CO_2_ in the atmosphere and an increase of the temperature gradient between low and high latitudes.

## Supporting information

Supporting Information S1

## Data Availability

The list the planktonic and benthic foraminiferal species, the distribution chart and absolute abundances of planktonic and benthic foraminifera, the distribution chart of calcareous nannofossils, and the bulk carbonate and foraminifera carbon and oxygen stable isotopes data are available in the Supporting  Information S1 and in the PANGAEA Data Publisher for Earth & Environmental Science at https://doi.pangaea.de/10.1594/PANGAEA.939392.
