# Supplementary material for: Late Cretaceous Paleoceanographic Evolution and the Onset of Cooling in the Santonian at Southern High Latitudes (IODP Site U1513, SE Indian Ocean)
Source: Paleoceanogr Paleoclimatol. 2022 Jan 20;37(1):e2021PA004353. doi: 10.1029/2021PA004353 (PMC9303530; doi:10.1029/2021PA004353)
Supplement: Supplementary file 1 — Supporting Information S1 [file PALO-37-0-s001.docx]

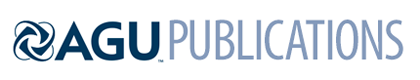


***Paleoceanography and Paleoclimatology***

Supporting Information for

**Late Cretaceous paleoceanographic evolution and the onset of cooling in the Santonian at southern high latitudes (IODP Site U1513, SE Indian Ocean)**

Maria Rose Petrizzo^1^, Kenneth G. MacLeod^2^, David K. Watkins^3^, Erik Wolfgring^1,4^, Brian T. Huber^5^

^1^*Dipartimento di Scienze della Terra “A. Desio”, Università degli Studi di Milano, via Mangiagalli 34, 20133 Milano, Italy, mrose.petrizzo@unimi.it, erik.wolfgring@guest.unimi.it*

^2^*Department of Geological Sciences, University of Missouri-Columbia, Columbia, MO 65211, USA, MacLeodK@missouri.edu*

^3^*Department of Earth and Atmospheric Sciences, University of Nebraska, Lincoln, NE 68588, United States, dwatkins1@unl.edu*

^4^*Department of Geology, University of Vienna, Vienna, Austria, erik.wolfgring@univie.ac.at*

^5^*National Museum of Natural History, Smithsonian Institution, MRC-121, Washington, DC 20013, huberb@si.edu*

**Contents of this file**

Text S1 to S2

References Text S1 to S2

**Introduction**

This study uses planktonic and benthic foraminiferal distribution and absolute abundance data and calcareous nannofossils distribution data. Bulk carbonate and foraminifera carbon and oxygen stable isotopes data are used to interpret the planktonic foraminifera paleoecology, the paleotemperatures and to document the carbon isotope record across the latest Cenomanian-Santonian interval. These data are available in the PANGAEA Data Publisher for Earth & Environmental Science at https://doi.pangaea.de/10.1594/PANGAEA.939392.

Text S1 and S2 list the planktonic and benthic foraminiferal species with authors and years mentioned in the text and in the distribution chart and illustrated in the figures.

**Text S1**

PLANKTONIC FORAMINIFERA- LIST OF SPECIES

Alphabetic list of planktonic foraminifera species with authors and years mentioned in the text and illustrated in Figs. 5-8. The specimens are deposited in the Collection of Micropalentology (Micro-unimi) at the Department of Earth Sciences “A. Desio”, University of Milan.

*Archaeoglobigerina cretacea* (d'Orbigny, 1840)

*Contusotruncana morozovae* (Vasilenko, 1961)

*Costellagerina bulbosa* (Belford, 1960)

*Costellagerina pilula* (Belford, 1960)

*Dicarinella hagni* (Scheibnerova, 1962)

*Dicarinella marginata* (Reuss, 1845)

*Falsotruncana maslakovae* Caron 1981

*Globigerinelloides alvarezi* (Eternod Olvera, 1959)

*Globigerinelloides asper* (Ehrenberg, 1854)

*Globigerinelloides prairiehillensis* Pessagno 1967

*Globigerinelloides ultramicrus* (Subbotina, 1949)

*Globigerinelloides yaucoensis* (Pessagno, 1960)

*Globotruncana arca* (Cushman, 1926a)

*Globotruncana bulloides* Vogler, 1941

*Globotruncana culverensis* Barr 1962

*Globotruncana hilli* Pessagno 1967

*Globotruncana linneiana* (d'Orbigny 1839)

*Globotruncana neotricarinata* Petrizzo, Falzoni, Premoli Silva 2011

*Liuenella falklandica* Georgescu 2008

*Marginotruncana coronata* (Bolli, 1945)

*Marginotruncana pseudolinneiana* Pessagno 1967

*Marginotruncana pseudomarginata* Neagu 2012

*Muricohedbergella crassa* (Bolli, 1959)

*Muricohedbergella delrioensis* (Carsey, 1926)

*Muricohedbergella planispira* (Tappan, 1940)

*Planoheterohelix globulosa* (Ehrenberg, 1840)

*Planoheterohelix papula* (Belford, 1960)

*Whiteinella baltica* Douglas & Rankin 1969

*Whiteinella brittonensis* (Loeblich & Tappan, 1961)

*Whiteinella paradubia* (Sigal, 1952)

**Text S2**

BENTHIC FORAMINIFERA- LIST OF SPECIES

Alphabetic list of benthic foraminifera species with authors and years mentioned in the text. The specimens are deposited in the Collection of Micropalentology (Micro-unimi) at the Department of Earth Sciences “A. Desio”, University of Milan.

*Anomalinoides* sp. Brotzen, 1942

*Ammodiscus cretaceous* Reuss, 1845

*Anomalinoides undulatus* Belford, 1960

*Aragonia velascoensis* Cushman, 1925

*Astacolus* spp. (juvenile) Montfort, 1808

*Astacolus jarvisii* Brotzen, 1936

*Bathysiphon* sp. Sars, 1872

*Cibicidoides* sp. Thalmann, 1939

*Cibicidoides* sp. 2 (*Cibicidoides* sp. cf. *C. voltzianus*) Thalmann, 1939

*Eponides* sp. A (Montfort, 1808)

*Cibicidoides* sp. cf. *C. perlucidus* Thalmann, 1939

*Cibicidoides* sp. A. Thalmann, 1939

*Citharina* sp. Thalmann, 1939

*Conorbina marginata* Brotzen, 1936

*Colomia cretacea* Cushman & Bermúdez, 1948

*Dentalina linearis* Roemer, 1841

*Dentalina cylindroides* Reuss, 1860

*Dentalina* sp. Risso, 1826

*Dentalina catenula* Reuss, 1860

*Laevidentalina legumen* (Reuss, 1845)

*Laevidentalina reflex*a (Morrow, 1934)

*Dorothia bulletta* (Carsey, 1926)

*Dorothia oxycona* (Reuss, 1860)

*Dorothia trochus* (d’Orbigny, 1840)

*Ellipsoglandulina* sp. Silvestri, 1900

*Ellipsoglandulina labiata* Silvestri, 1903

*Ellipsoglandulina ovata* Gabor-Biedowa, 1992

*Ellipsodimorphina* sp. Silvestri, 1901

*Ellipsoidella* sp. Heron-Allen & Earland, 1910

*Polymorphina* sp. A Roemer, 1841

*Ellipsoidella primitiva* (Cushman, 1933)

*Ellipsoidella binaria* Belford, 1960

*Ellipsoidella divergens* Storm, 1929

*Ellipsoidella pleurostomides* Heron-Allen & Earland, 1910

*Eponides* sp. Montfort, 1908

*Fissurina alata* Reuss, 1851

*Frondicularia* sp. d’Orbigny, 1826

*Frondicularia mucoronata* Reuss, 1845

*Gaudryina pyramidata* Cushman, 1926b

*Gavelinella* spp. Brotzen, 1942

*Gavelinella* sp. cf. *G. stellula* Belford, 1960

*Gavelinella eriksdalensis* Brotzen, 1942

*Gavelinella* *insculpta* Belford, 1960

*Gavelinella stephensoni* Cushman, 1938

*Gavelinella compressa* Sliter, 1968

*Gavelinella* sp. aff. *dani* Brotzen, 1942

*Gavelinella* cf. *costata* Brotzen, 1942

*Glandulina bicamerata* Hermann, 1917

*Glandulina* sp. d'Orbigny, 1839

*Goloborotalites conicus* (Carsey, 1926)

*Globorotalites spineus* (Cushman, 1926a)

*Globorotalites michelinianus* (d'Orbigny, 1840)

*Globorotalites* sp. cf. *G. subconicus* (Morrow, 1934)

*Globulimina lacrima* (Reuss, 1845)

*Gavelinella stephensoni* (Cushman, 1938)

*Gyroidinoides nitidus* (Reuss ?)

*Gyroidinoides globosus* Hagenow 1842

*Gyroidinoides praeglobosa* Brotzen, 1936

*Gyroidinoides quadratus* (Cushman & Church, 1929)

*Gyroidinoides exsertus* (=*Gyroidina exserta*) Belford, 1960

*Gyroidinoida noda* Belford, 1960

*Gyroidinoides diversus* Belford, 1960

*Gyroidinoides girardianus* (Reuss, 1851)

*Gyroidinoides* sp. d'Orbigny, 1826

*Gyroidinoides michelinianus* (d'Orbigny, 1840)

*Guttulina* sp. d'Orbigny, 1839

*Laevidentalina* sp. cf. *D. catenula* (Reuss, 1860)

*Laevidentalina* sp. Loeblich & Tappan, 1986

*Laevidentalina* sp. cf. *L. gracilis* Loeblich & Tappan, 1986

*Lagena apiculata* Reuss (1851)

*Lagena emaciata* Reuss (1863)

*Lagena* sp. Walker & Jacob, 1798 in Kanmacher, 1798

*Lenticulina acutauricula* (Fichtel & Moll, 1798)

*Lenticulina macrodiscus* (Reuss, 1863)

*Lenticulina* sp. Lamarck (1804)

*Lenticulina muensteri* (Roemer, 1839)

*Lenticulina* sp. cf. *Vonderschmitti* Reichel, 1946

*Lenticulina* sp. cf. *L. exrata* Lamarck, 1804

*Lenticulina* sp.1 Lamarck, 1804

*Lenticulina nodosa* Reuss, 1863

*Lenticulina* sp. 2 Lamarck, 1804

*Lituotuba* sp. Rhumbler, 1895

*Marginulinopsis* sp. Silvestri, 1904

*Marginulina* sp. d'Orbigny, 1826

*Marginulina bullata* Reuss, 1845

*Marginulina* sp. 1 (sensu Quilty 1992) d'Orbigny, 1826

*Marginulina* sp. cf. *M. tichae* d'Orbigny, 1826

*Marginulina stephensoni* (sensu Basov & Krasheninnikov 1983) Cushman, 1937

*Nodosaria* sp. Lamarck, 1816

*Nodosaria prismatica* Reuss, 1860

*Nodosaria aspera* Reuss, 1845

*Notoplanulina* sp. A (*N.* sp. sensu Quilty 1992) (Finlay, 1939)

*N. rakauroana* (Finlay, 1939)

*Notoplanulina* sp. Malumián & Masiuk, 1976

*Nuttallinella coronula* (Belford, 1958)

*N. florealis* (White, 1928)

*Nuttallinella* sp. Belford, 1959

*Nodosaria* sp. Lamarck, 1816

*Nodosaria limbata* d'Orbigny, 1840

*Nodoraria prismatica* Reuss, 1860

*Oolina* spp. d'Orbigny, 1839

*Oolina* sp. cf. *O. simplex* Reuss, 1851

*Oridorsalis primitivus* Kaiho, 1998

*Oridorsalis umbonatus* Reuss, 1851

*Osangularia lens* Brotzen, 1940

*Osangularia* sp. Brotzen, 1940

*Paralabamina hillebrandti* (Fisher, 1969)

*Planularia* sp. Defrance 1826

*Pleurostomella* sp. Reuss, 1860

*Pleurostomella copiosa* Bukalova, 1960

*Pleurostomella obtusa* Berthelin, 1880

*Praebulimina* sp. Hofker, 1953

*Praebulimina reussi* (Morrow, 1934)

*Praebulimina kickapoosensis* Cole, 1938

*Pseudouvigerina* sp. Cushman, 1927

*Pullennia americana* Cushman, 1936

*Pullenia jarvisii* Cushman, 1936

*Quadrimorphina* sp. Finlay, 1939

*Quadrimorphina allomorphinoides* (Reuss, 1860)

*Quadrimorphina camerata* (Brotzen, 1936)

*Quadrimorphina* sp. A (sensu Bolli et al. 1994) Finlay, 1939

*Ramulina* sp. cf. *R. wright* Brady, 1879

*Reussella szajnochae* (Grzybowski, 1896)

*Reophax subfusiformis* Earland, 1933

*"Rhabdammina"* sp. (tubular agglutinated foraminifera, gen. indet) Sars in Carpenter, 1869

*Saracenaria* sp. Defrance, 1824

*Saracenaria navicula* (d'Orbigny, 1840)

*Siphonodosaria* sp. Silvestri, 1924

*Sigmoilina* sp. (Brady, 1884)

*Stilostomella plummerae* Cushman, 1940

*Valvulineria muchisonensis* Belford, 1960

*Valvulineria erugata* Belford, 1960

*Valvulineria* sp. Cushman, 1926b

*Vaginulina* sp. d'Orbigny, 1826

*Recurvoides* sp. (Brady, 1881)

**References Text S1 and Text S2**

Barr, F.T. (1962). Upper Cretaceous planktonic foraminifera from the Isle of Wight, England. *Palaeontology* 4(4), 552-580.

Basov, I. A., Krasheninnikov, V. A. (1983). Benthic foraminifers in Mesozoic and Cenozoic sediments of the southerwestern Atlantic as an indicator of paleoenvironment, Deep Sea Drilling Project Leg 71. In Ludwig, W. J., Krasheninnikov, V. A., Basov, I. A., Bayer, U., Bloemendal, J., Bornhold, B. et al., *Initial Reports of the Deep Sea Drilling Project* 71, 739-787, Washington (U.S. Government Printing Office).

Belford, D.J. (1958). The genera *Nuttallides* Finlay, 1939, and *Nuttallina*, n. gen. *Contributions from the Cushman Foundation for Foraminiferal Research* 9, 93-98.

Belford, D.J. (1959). *Nuttallinella*, new name for *Nuttallina* Belford, 1958 (non *Nuttallina* Dall, 1871). *Contributions from the Cushman Foundation for Foraminiferal Research* 10, 20.

Belford, D.J. (1960). Upper Cretaceous foraminifera from the Toolonga Calcilutite and Gingin Chalk, Western Australia. *Australia Bureau of Mineral Resources, Geology and Geophysics Bulletin* 57, 1-198.

Berthelin, G. (1880). Mémoire sur les Foraminifères fossiles de l'Etage Albien de Moncley (Doubs). *Mémoires de la Société Géologique de France*, (3), ser. 3 31, 1-84.

Bolli, H.M. (1945). Zur Stratigraphie der oberen Kreide in den höheren helvetischen Decken. *Eclogae Geologicae Helvetiae* 37, 217-328.

Bolli, H.M. (1959). Planktonic foraminifera from the Cretaceous of Trinidad, B. W. I. *Bulletins of American Paleontology* 39(179), 253-277.

Bolli, H.M., Beckmann, J.P., Saunders, J.B. (1994). *Benthic foraminiferal biostratigraphy of the southern Caribbean region*. Cambridge University Press.

Brady, H.B. (1879). Notes on some of the Reticularian Rhizopoda of the "Challenger" Expedition. II. Additions to the knowledge of porcellanous and hyaline types. *Quarterly Journal of Microscopical Science* 19 (75), 261-299.

Brady, H.B. (1881). Notes on some of the Reticularian Rhizopoda of the "Challenger" Expedition. Part III. *Quarterly Journal of Microscopical Science* (2) 21 (81), 31-71.

Brady, H.B. (1884). Report on the Foraminifera dredged by H.M.S. Challenger during the Years 1873-1876. Report on the Scientific Results of the Voyage of H.M.S. Challenger during the years 1873–76. *Zoology* 9 (part 22), i-xxi, 1-814; pl. 1-115.

Brotzen, F. (1936). Foraminiferen aus dem schwedischen untersten Senon von Eriksdal in Schonen. *Arsbok Sveriges Geologiska Undersökning ser* C. 30(3), 1-206.

Brotzen, F. (1940). Flintrännans och Trindelrännans geologi (Öresund). *Årsbok Sveriges Geologiska Undersökning* 34(5), 1-33.

Brotzen, F. (1942). Die Foraminiferengattung *Gavelinella* nov. gen. und die Systematik der Rotaliiformes. *Sveriges Geologiska Undersökning* 36(8) C (451), 1-60.

Bukalova, G.V. (1960). Булиминиды и эллипсоидиниды альбских отложений междуречья Белой и Кубани (Северное Предкавказье) - Buliminids and ellipsoidinids of the Albian deposits of the Belaya and Kuban interfluve (Northern Ciscaucasia). Тр. ВНИГНИ, Палеонт. сб. - *Trudy VNIGNI, Pal. Sbornik* 16(3), 225-234.

Caron, M. (1981). Un nouveau genre de Foraminifere planctonique du Cretace: *Falsotruncana* nov. gen. *Eclogae Geologicae Helvetiae* 74(1), 65-73.

Carpenter, W.B. (1869). On the Rhizopodal Fauna of the Deep Sea. *Proceedings of the Royal Society of London* 18(114-122), 59-62.

Casey, D.O. (1926). Foraminifera of the Cretaceous of central Texas. *University of Texas Bulletin* 2612, 1-56.

Cole, W.S. (1938). Stratigraphy and micropaleontology of two deep wells in Florida. *Florida State Geological Survey, Geological Bulletin* 16, 1-73.

Cushman, J.A. (1925). Some new Foraminifera from the Velasco Shale of Mexico. *Contributions from the Cushman Laboratory for Foraminiferal Research* 1, 18-23

Cushman, J. A. (1926a). Some foraminifera from the Mendez shale of Eastern Mexico. *Contributions from the Cushman Laboratory for Foraminiferal Research* 2(1), 16-24.

Cushman, J.A. (1926b). The foraminifera of the Velasco Shale of the Tampico Embayment. *Bulletin of the American Association of Petroleum Geologists* 10(6), 581-612.

Cushman, J.A. (1927). Some new genera of the Foraminifera. *Contributions from the Cushman laboratory for foraminiferal research* 2(4), 77-81.

Cushman, J.A. (1933). New American Cretaceous Foraminifera. *Contributions from the Cushman laboratory for foraminiferal research* 9(3), 49-64.

Cushman, J.A. (1936). Part IV. Cretaceous and Late Tertiary foraminifera. *Bulletin of the Geological Society of America*, 47(3), 413-440.

Cushman, J.A. (1937). Some notes on Cretaceous species of *Marginulina*. *Contributions from the Cushman laboratory for foraminiferal research* 13(4), 91-99.

Cushman, J.A. (1938). Some new species of rotaliform Foraminifera from the American Cretaceous. *Contributions from the Cushman laboratory for foraminiferal research* 14(3), 66-71.

Cushman, J. A. (1940). Midway Foraminifera from Alabama. *Contributions from the Cushman Laboratory for Foraminiferal Research*, 16(3): 51-73.

Cushman, J.A., & Church, M.A. (1929). Some Upper Cretaceous Foraminifera from near Coalinga, California. *Proceedings of the California Academy of Sciences* (4) 18 (16), 497-530.

Cushman, J.A., & Bermúdez, P.J. (1948). *Colomia*, a new genus from the Upper Cretaceous of Cuba. *Contr. Cushman Lab. Foram. Res* 24, 12, 13

d'Orbigny, A. (1839). *Foraminifères*. A. Bertrand Editeur, Paris, 1-224.

d'Orbigny, A. (1840). Mémoire sur les foraminiféres de la craie blanche du bassin de Paris. *Mémoires de la Société Géologique de France* 4(1), 1-51.

Defrance, J.L.M. in Blainville. (1824). Dictionnaire des Sciences Naturelles. F.G. Levrault, Strasbourg, vol. 32.

Defrance, J.L.M. in Blainville (1826). Dictionnaire des Sciences Naturelles. F.G. Levrault, Strasbourg, vol. 41.

Douglas, R.G., & Rankin, C. (1969). Cretaceous planktonic foraminifera from Bornholm and their zoogeographic significance. *Lethaia* 2, 185-217.

Earland, A. (1933). Foraminifera, Part II, South Georgia. *Discovery Reports* 7, 27-138 + plates.

Ehrenberg, C.G. (1840). Uber die Bildung der Kreidenfelsen und des Kreidemergels durch unsichtbare Organismen*.* *Physik. Abh.* (1838), Berlin, 59-147.

Ehrenberg, C.G. (1854). Mikrogeologie: Das Erden und Felsen schaffende Wirken des unsichtbar kleinen selbständigen Lebens auf der Erde*.* Leopold Voss, Leipzig, 1-374.

Eternod Olvera, Y. (1959). Foraminíferos del Cretácico Superior de la cuenca de Tampico-Tuxpan, Mexico. *Boletin de la Asociación Mexicana de Geologos Petroleros* 11, 61-134.

Fichtel, L.v., & Moll, J.P.C. (1798). Testacea microscopia, aliaque minuta ex generibus Argonauta et Nautilus, ad naturam delineata et descripta. A. Pichler, Wien. xii + 123 pp., 24 pl., p. 103, pl. 19 b, c.

Finlay, H.J. (1939). New Zealand Foraminifera: Key Species in Stratigraphy - No. 3. *Transactions of the Royal Society of New Zealand* 69, 309-329.

Fisher, M.J. 1969 Benthonic Foraminifera from the Maestrichtian chalk of the Galicia Bank, west of Spain. *Palaeontology* 12, 189-200.

Gawor-Biedowa, E. (1992). New foraminifera of the Campanian and Maastrichtian in the Lublin Region (Eastern Poland). *Kwartalnik Geologiczny* 36(1), 75-96.

Georgescu, M.D. (2008). A new planktonic foraminifer (Family Hedbergellidae Loeblich and Tappan, 1961) from the lower Campanian sediments of the Falkland Plateau, South Atlantic Ocean (DSDP Site 511). *Journal of Foraminiferal Research* 38, 157-161.

Georgescu, M.D. (2010). *Liuenella*, new name for *Liuella* Georgescu, 2008. *Journal of Foraminiferal Research* 40, 206.

Grzybowski, J. (1896). Otwornice czerwonych ilow z Wadowic. Rozprawy, Akademia Umiejetnosci w Krakowie, Wydzial Matematyczno-Przyrodniczy, Kraków.

von Hagenow, K.F. (1842). Monographie der Rügen'schen Kreide-Versteinerungen. III Abtheilung: Mollusken. *Neues Jahrbuch für Mineralogie, Geognosie, Geologie und Petrefaktenkunde* 528-575, pl. 9.

Heron-Allen, E., & Earland, A. (1910). On the recent and fossil Foraminifera of the shore-sands of Selsey Bill, Sussex - V. The Cretaceous Foraminifera. *Journal of the Royal Microscopical Society* 401-426.

Herrmann, A. (1917). Vierter Beitrag zur Kenntnis des Vorkommens von Foraminiferen im Tertiär des Unterelsass. *Mitteilungen der Geologischen Landesanstalt von Elsass-Lothringen* 10, 1-286.

Hofker, J. (1953). Types of Genera Described in Part III of the "Siboga Foraminifera". *The Micropaleontologist* 7(1), 26-28.

Kaiho, K. (1998). Phylogeny of Deep-Sea Calcareous Trochospiral Benthic Foraminifera: Evolution and Diversification. *Micropaleontology* 44(3), 291.

Kanmacher F. (1798). Essays on the microscope. The Second Edition, with considerable additions and improvements. Dillon & Keating, London. xvii + [7 unnumbered] + 724 pp., 32 pl.

Lamarck, J.B.P.A. de. (1804). Suite des mémoires sur les fossiles des environs de Paris. *Annales du Muséum d'Histoire Naturelle* 5, 28-36; 91-98; 179-188; 237-245; pl. 59-62 [plates published 1806 in vol. 8].

Lamarck, J. B. P. A. de M. de. (1816). Tableau encyclopédique et méthodique des trois règnes de la nature, Mollusques et polypes divers. Part 23 [Livraison 84, 14 December 1816], Tome 3, pp. 1-16, pls. 391-431, 431 bis, 431 bis*, 432-488, Paris: Vve Agasse.

Loeblich, A.R., & Tappan, H. (1961). Cretaceous planktonic foraminifera: Part I-Cenomanian. *Micropaleontology* 7, 257-304.

Loeblich, A.R., Tappan, H. (1986). Some New and Revised Genera and Families of Hyaline Calcareous Foraminiferida (Protozoa). *Transactions of the American Microscopical Society* 105(3), 239-265.

Malumián, N., & Masiuk, V. (1976). Foraminiferos de la formacion Cabeza de Leon (Cretacio Superior, Tierra del Fuego, República Argentinia). *Revista Asocoación Geológica Argentinia* 31, 3, 180-202.

Montfort P. (1808-1810). Conchyliologie systématique et classification méthodique des coquilles. Paris: Schoell. Vol. 1: pp. lxxxvii + 409 [1808]. Vol. 2: pp. 676

Morrow, A.L. (1934). Foraminifera and ostracoda from the Upper Cretaceous of Kansas. *Journal of Paleontology* 8, 186-205.

Neagu, T. (2012). *“Rosalina” marginata* Reuss 1845 (Foraminifera) and its taxonomic position. *Studia UBB Geologia* 57, 35-38.

Orbigny, A.D.d'. (1826). Tableau méthodique de la classe des Céphalopodes. *Annales des Sciences Naturelles* vol. 7: 96-169, 245-314.

Orbigny, A.D.d'. (1839). Foraminifères, in de la Sagra R., Histoire physique, politique et naturelle de l'ile de Cuba. A. Bertrand, 1-224.

Orbigny, A.D.d'. (1840). Mémoire sur les foraminifères de la craie blanche du bassin de Paris. *Mémoires de la Société géologique de France. 1ère série*, tome IV, mémoire n° 1.

Pessagno, E.A. (1960). Stratigraphy and micropaleontology of the Cretaceous and lower Tertiary of Puerto Rico. *Micropaleontology* 6(1), 87-110.

Pessagno, E.A. (1967). Upper Cretaceous planktonic foraminifera from the western Gulf Coastal Plain. *Palaeontographica Americana* 5, 245-445

Petrizzo, M.R., Falzoni, F., & Premoli Silva, I. (2011). Identification of the base of the lower-to-middle Campanian *Globotruncana ventricosa* Zone: Comments on reliability and global correlations. *Cretaceous Research* 32, 387-405.

Quilty, P.G. (1992). Upper Cretaceous planktonic foraminifera and biostratigraphy. In *Proceedings of the Ocean Drilling Program, Scientific Results* 120, 371-392. College Station, TX: Ocean Drilling Program.

Reichel, M. (1946). Sur quelques foraminifères nouveaux du Permien méditerranéen. *Eclogae Geologicae Helvetiae* 38(2)[1945], 524-560.

Reuss, A.E. (1845). Die Versteinerungen der böhmischen Kreideformation. E. Schweizerbart’sche Verlagsbuchhandlung und Druckerei, Stuttgart.

Reuss, A.E. (1845a). *Die Versteinerungen der böhmischen Kreide-formation.* E. Schweizebart, Stuttgart, 1-58.

Reuss, A.E. (1851). Über die fossilen Foraminiferen und Entomostraceen der Septarienthone der Umgegend von Berlin. *Zeitschrift der Deutschen Geologischen Gesellschaft*, 3 (1), 49-92.

Reuss, A.E. (1860). Die Foraminiferen der westphälischen Kreideformation. *Sitzungsberichte der mathematisch-naturwissenschaflichen Classe der kaiserlichen Akademie der Wissenschaften*, 40 (8), 147-238.

Reuss, A.E. (1863). Die Foraminiferen - Familie der Lagenideen. *Sitzungsberichte der Akademie der Wissenschaften. Mathematisch-naturwissenschaftliche Klasse, Wien*, 46, 308-342.

Rhumbler, L. (1895). Entwurf eines natürlichen Systems der Thalamophoren. *Nachrichten der Gesellschaft der Wissenschaften zu Göttingen, Mathematisch-Physikalische Klasse*, 1895, 51-98.

Risso, A. (1826-1827). Histoire naturelle des principales productions de l'Europe Méridionale et particulièrement de celles des environs de Nice et des Alpes Maritimes. Paris, F.G. Levrault. 3(XVI): 1-480, 14 pls.

Roemer, F.A. (1839). Die Versteinerungen des norddeutschen Oolithengebirges, ein Nachtrag. Hahn, Hannover, 1-59.

Römer, F.A. (1841). Die Versteinerungen des Norddeutschen Kreidegebirges. Mit sechszehn lithographirten Tafeln. Hahn'schen, Hofbuch-handlung, Hannover, [1840] iv + 49-145, pls. 8-20.

Sars, G.O. (1872). Undersøgelser over Hardangerfjordens fauna I. *Förhadlingar i Videnskabsselskabet i Christiania* 1871, 246-286.

Scheibnerová, V. (1962). Stratigrafia strednej a vrchnej kriedy tetydní oblasti na zaklade globotruncanid - Stratigraphy of the Middle and Upper Cretaceous of the Tethyan region on the basis of the Globotruncanids. *Geologiscky Zbornik -Geologica Carpathica* 13(2), 219-226.

Sigal, J. (1952). Apercu stratigraphique sur la micropaleontologie du Cretace. *XIX Congr. géol. internat., Algers, Monographies Regionales* 1(26), 3-43.

Silvestri, A. (1900). Sur genere *Ellipsoglandulina*. *Atti e Rendiconti R. Accademia di Scienze, Lettere ed Arte degli Zelanti, Acireale, Cl. Sci. Mem.* 10, 1-9.

Silvestri, A. (1901). Sulla struttura di certe Polimorfine dei dintorni di Caltagirone. *Bollettino Accademia Gioenia di Scienze Naturali, Catania* n. ser. 69, 14-18.

Silvestri, A. (1903). Alcune osservazioni sui Protozoi fossili piemontesi. *Atti Reale Accademia delle Scienze di Torino* 38(6), 206-217.

Silvestri, A. (1904). Ricerche strutturali su alcune forme dei Trubi dei Bonfornello (Palermo). *Memorie dell'Accademia Pontificia dei Nuovi Lincei* 22, 235-276.

Silvestri, A. (1924). Fauna Paleogenica di Vasciano presso Todi. *Bollettino della Società Geologica Italiana* 42, 7-29.

Sliter, W.V. (1968). Upper Cretaceous Foraminifera from southern California and northwestern Baja California, Mexico. *University of Kansas Paleontological Contributions* 49(7), 1-141.

Storm, H. (1929). Zur Kennthis der Foraminiferenfauna im Oberturon und Emscher der Böhmischen Kreideformation. *Lotos, Prag, Tschechoslowakei* 77, 39-62.

Subbotina, N.N. (1949). Микрофауна меловых отложений южного склона Кавказа [Microfauna from the Cretaceous of the southern slope of the Caucasus]. *VNIGRI, Microfauna of the oil fields of the USSR* 2, 34, 5-36.

Tappan, H. (1940). Foraminifera from the Grayson formation of Northern Texas. *Journal of Paleontology* 14(2), 93-126.

Thalmann, H.E. (1939). Bibliography and index to new genera, species and varieties of foraminifera for the year 1936. *Journal of Paleontology* 13, 425-465.

Vasilenko, V.P. (1961). Upper Cretaceous foraminifera of the Mangyshlak Peninsula. *Trudy Vsesoyuznego Neftyanogo Nauchno-Issledovatel 'skogo Geologo-Razvedochnogo Instituta (VNIGRI)* 171, 160-161.

Vogler, J. (1941). Ober-Jura und Kreide von Misol. *In*, Boehm, G. & Wanner, J. (eds) *Beitrage zur Geologie von Niederlandisch-Indian,* 243-293.

White, M.P. (1928). Some index foraminifera of the Tampico Embayment area of Mexico. Parts 1, 2. *Journal of Paleontology* 2, 3-4, 177-215, 280-317.
